# Specific gut microbes are associated with the incidence of cardiometabolic disease in the HELIUS cohort

**DOI:** 10.1038/s41522-026-00952-6

**Published:** 2026-03-07

**Authors:** Barbara J. H. Verhaar, Thomas A. Bouwmeester, Henrike Galenkamp, Bert-Jan H. van den Born, Max Nieuwdorp

**Affiliations:** 1 Department of Vascular Medicine, Amsterdam UMC, Amsterdam, The Netherlands; 2 Amsterdam Cardiovascular Sciences, Amsterdam UMC, University of Amsterdam, Amsterdam, The Netherlands; 3 Amsterdam Gastroenterology Endocrinology Metabolism, Amsterdam UMC, University of Amsterdam, Amsterdam, The Netherlands; 4 Department of Public and Occupational Health, Amsterdam UMC, Amsterdam, The Netherlands; 5 Amsterdam Public Health Research Institute, Amsterdam UMC, Amsterdam, The Netherlands; 6 Department of Experimental Vascular Medicine, Amsterdam UMC, Amsterdam, The Netherlands

**Keywords:** Cardiology, Diseases, Medical research, Microbiology

## Abstract

Associations between the gut microbiota and cardiometabolic health are well established, but evidence from longitudinal studies remains limited. In the prospective multi-ethnic HELIUS cohort, we investigated whether baseline gut microbiota composition was associated with long-term cardiometabolic outcomes. Fecal samples from 4792 participants were collected at baseline and analyzed using 16S rRNA sequencing. At follow-up, new diagnoses of hypertension, dyslipidemia, and diabetes were assessed, and major adverse cardiovascular events (MACE and MACE + , including angina pectoris) were obtained from hospital and mortality registries. Logistic regression was used to study associations with incident cardiometabolic disease, while Cox regression evaluated associations with MACE among participants without cardiovascular disease at baseline. During follow-up, 129 participants experienced MACE (2.7%) and 180 MACE+ (3.8%). Higher abundance of *Eubacterium xylanophilum* group spp. and *Akkermansia muciniphila* was associated with lower MACE+ risk, whereas *Ruminococcus gnavus* group spp. was associated with higher MACE risk, although only *Eubacterium xylanophilum* group spp. remained significant after full adjustment. Several taxa were associated with incident cardiometabolic disease, and exploratory metabolomics linked *Ruminococcus gnavus* group spp. to bile acid and acylcarnitine metabolites. These findings suggest that gut microbiota composition is longitudinally associated with cardiometabolic disease.

## Introduction

The human gut microbiota has been extensively linked to various health outcomes, particularly in cardiovascular and metabolic health^1^. Mechanistic insights from animal studies and fecal microbial transplant trials underscore its role in driving insulin resistance and atherosclerosis^2^, while clinical trials with short-chain fatty acids—key microbiota-derived gut metabolites—have demonstrated modulating effects on appetite, glucose metabolism, and blood pressure^3,4^. However, most evidence to date remains cross-sectional, leaving the long-term effects of microbiota composition and function on human health largely unexplored.

Previous analyses in the multi-ethnic HELIUS cohort revealed ethnic differences in gut microbiota composition, which correlated with cardiometabolic outcomes^5–7^. In addition, a Mendelian randomization analysis provided valuable insights into the associations between genetic variants, microbiota differences, and cardiometabolic factors^8^. To further support a causal role of gut microbiota in cardiometabolic health, it is important to establish a temporal sequence of events, necessitating a considerable follow-up time. Previous research suggests that the gut microbiome remains relatively stable in adulthood^9^, influenced primarily by diet and health status^10^, making it a promising predictor of long-term outcomes.

Using follow-up data of the HELIUS cohort, we aimed to investigate the impact of baseline gut microbiota composition on long-term cardiometabolic outcomes. We hypothesized that previously reported cross-sectional associations between gut microbes and both blood pressure^6^ and lipid profiles^11^ translate to a higher incidence of major adverse cardiovascular events (MACE), and incident diabetes, dyslipidaemia, and hypertension. To test this, we leveraged follow-up data of the HELIUS multi-ethnic cohort, including data on new-onset cardiometabolic diagnoses, and additionally linked HELIUS data to population and hospital registries in order to perform survival analyses.

## Results

### Population characteristics

**Fig. 1:**
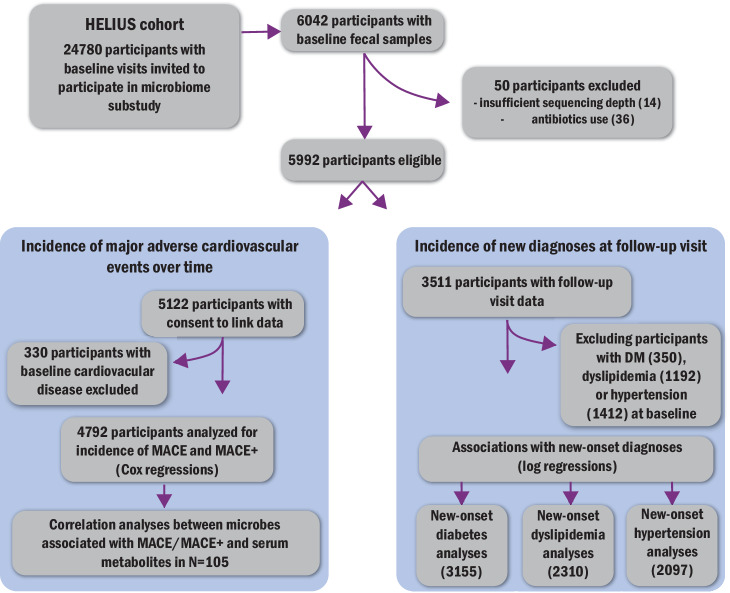
Study flowchart. Study flowchart describing the selection of participants in different parts of the project. MACE major adverse cardiovascular events. MACE + = MACE, including hospitalizations for angina pectoris. DM diabetes mellitus.

Of the 6028 participants of the HELIUS cohort with baseline microbiome data, we included 4792 participants without antibiotic use and with consent for mortality and hospital registry data linkage (Fig. 1). These participants had a mean age of 49.7 ± 11.7 years, of whom 2528 (52.8%) were women (Table 1). The largest ethnic group was Dutch (*N* = 1526), followed by African Surinamese (*N* = 1243) and South-Asian Surinamese (*N* = 740). The follow-up period until censoring, defined as either the end of follow-up or a MACE event, was 9.5 ± 1.6 years. During follow-up, 129 participants (2.7%) reached a MACE endpoint, including 22 cardiovascular deaths (0.5%) and 107 non-fatal cardiovascular events (2.2%). Ethnic stratification (Supplementary Table 1) revealed the highest MACE incidence in the South-Asian Surinamese group (5.0%). The total number of MACE+ events, additionally including angina pectoris, cardiac arrhythmia, and heart failure, was 180 (3.8%). Similar ethnic differences were observed, with the highest incidence again in the South-Asian Surinamese group (8.4%). Non-cardiovascular deaths occurred in 169 participants (3.5%).

**Table 1:** HELIUS cohort characteristics

	Baseline data of all participants with microbiome and registry data	Baseline data of participants with follow-up visits	Follow-up data of participants with follow-up visits
*n*	4792	3511	3511
Age, years	49.7 ± 11.7	51.0 ± 10.8	57.2 ± 11.0
Women	2528 (52.8)	1863 (53.1)	-
Ethnicity			
Dutch	1433 (29.9)	1149 (32.7)	-
South-Asian Surinamese	643 (13.4)	530 (15.1)	-
African Surinamese	1160 (24.2)	863 (24.6)	-
Ghanaian	469 (9.8)	268 (7.6)	-
Turkish	415 (8.7)	228 (6.5)	-
Moroccan	573 (12.0)	418 (11.9)	-
Other ethnicities	99 (2.1)	55 (1.6)	-
Follow-up time, years	9.5 ± 1.6	-	6.2 ± 1.2
BMI, kg/m^2^	27.2 ± 4.9	26.9 ± 4.7	27.4 ± 4.8
Current smoking	958 (20.2)	628 (18.0)	548 (15.6)
Alcohol consumption			
low	3432 (72.6)	2483 (71.1)	2337 (78.6)
moderate	936 (19.8)	751 (21.5)	515 (17.3)
high	360 (7.6)	260 (7.4)	123 (4.1)
Diabetes	532 (11.1)	350 (10.0)	500 (14.3)
Hypertension	1941 (40.6)	1412 (40.2)	1597 (45.5)
Dyslipidemia	998 (20.1)	1192 (34.0)	1247 (35.7)
Systolic BP, mmHg	129.7 ± 18.3	129.1 ± 17.6	129.2 ± 17.9
Diastolic BP, mmHg	81.1 ± 10.7	80.6 ± 10.2	78.7 ± 10.3
Total cholesterol, mmol/L	5.1 ± 1.0	5.1 ± 1.0	5.2 ± 1.1
LDL, mmol/L	3.1 ± 0.9	3.2 ± 0.9	3.2 ± 1.0
Triglycerides, mmol/L	0.9 [0.6, 1.3]	0.9 [0.6, 1.2]	1.0 [0.4, 2.1]
HbA1c, mmol/mol	39.4 ± 8.7	39.3 ± 7.7	40.1 ± 9.4
MACE	129 (2.7)	-	-
Myocardial infarction or revascularization	62 (48.1)	-	-
Stroke	45 (34.9)	-	-
Cardiovascular death	22 (17.1)	-	-
MACE+	180 (3.8)	-	-

Data is presented as mean ± SD, median [interquartile range], or *n* (%).

*BMI* body mass index, *BP* blood pressure, *LDL* low-density lipoprotein, *MACE* major adverse cardiovascular event, *MACE+* major adverse cardiovascular event plus angina pectoris.

Of the 6028 baseline participants, 3511 attended a follow-up visit. Participants with follow-up visits were on average 2.9 years older, had lower BMIs, and were less often smokers than participants that were lost to follow-up (Supplementary Table 2). Their blood pressure, cholesterol levels, and HbA1c were comparable in absolute numbers, albeit significantly different due to the sample size. Over an average follow-up of 6.2 ± 1.2 years (Table 1**)**, there were 183 new cases of diabetes (5.8%), 375 new cases of dyslipidemia (18.6%), and 376 new cases of hypertension (20.8%).

### Gut microbiota composition and major adverse cardiovascular events

**Fig. 2:**
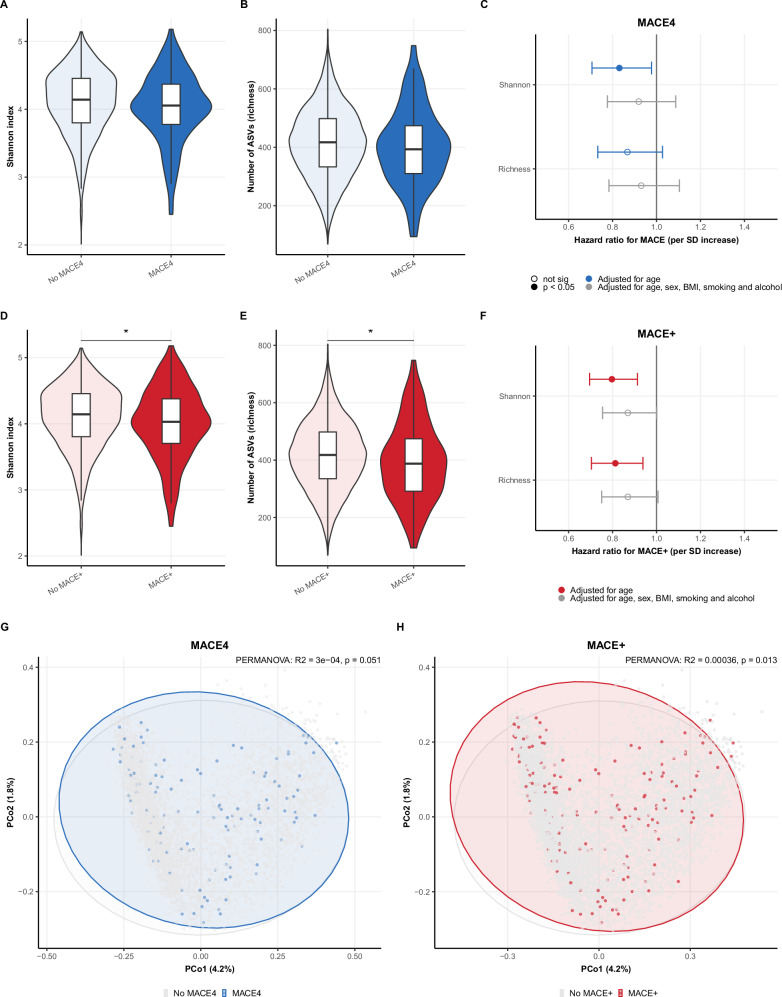
Microbial diversity and MACE. MACE (**A**–**C**) and MACE+ (**D**–**F**) and baseline diversity in the gut microbiome of 4792 participants. Alpha diversity indices, including Shannon index (**A**, **D**), and richness (**B**, **E**); differences tested with Mann–Whitney U tests. Cox regression models between alpha diversity metrics and MACE or MACE+ are shown in forest plots (**C**, **F**). Beta diversity as measured with Bray-Curtis distances is shown in principle coordinate analyses plots (**G**, **H**), differences tested with permuted analysis of variance (PERMANOVA). * *p* < 0.05.

To investigate associations between gut microbiota and MACE, we used mortality and hospital registry data. We first looked at differences in alpha and beta diversity between participants with and without MACE and MACE+ (Fig. 2). Age-adjusted Cox regressions revealed significant associations with alpha diversity as measured with the Shannon index and lower MACE (HR 0.83 (0.71–0.98) per SD increase, *p* = 0.025) and MACE+ (HR 0.80 (0.71–0.90), *p* = 0.001), and richness and MACE+ (HR 0.81 (0.70–0.93), *p* = 0.004). These associations lost significance after adjustment for sex, BMI, smoking, and alcohol. Beta diversity differences between participants were only significant for MACE+ (PERMANOVA *p* = 0.013), but with a negligible difference between groups.

**Fig. 3:**
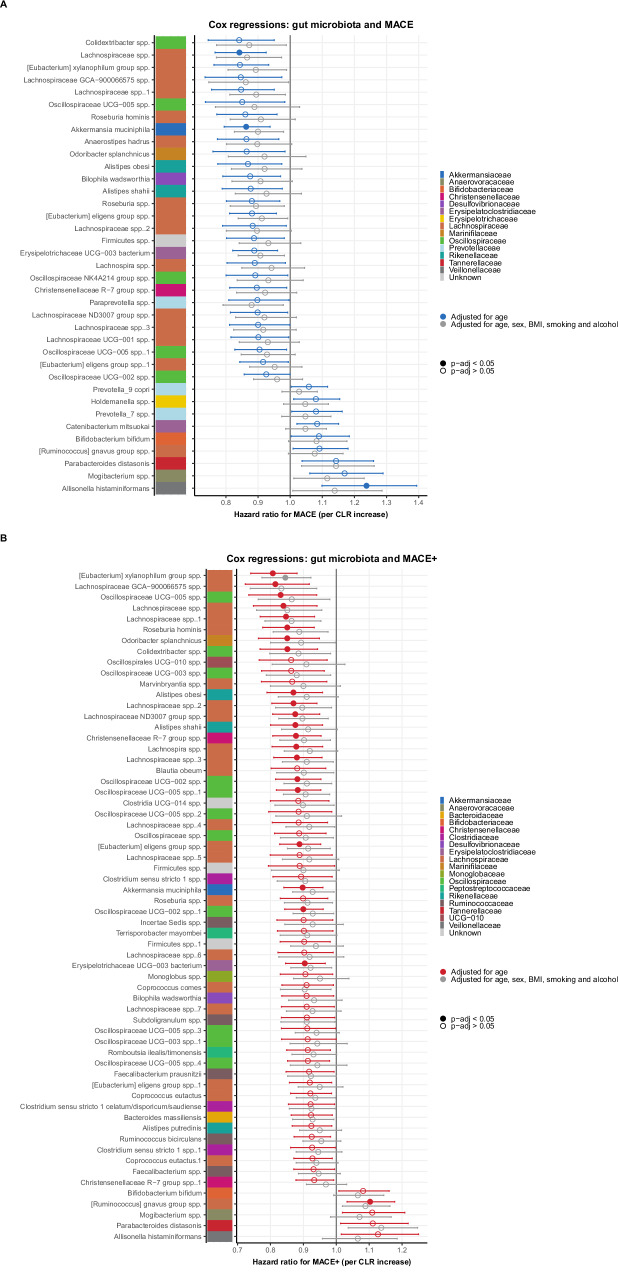
Cox regression models: baseline gut microbes and MACE. Age-adjusted regression models per outcome: MACE (**A**) and MACE+ (additionally including hospitalizations for angina pectoris; (**B**) in 4792 participants. Forest plots with estimates (hazard ratios) and 95% confidence intervals per centered log-ratio increase in the amplicon sequence variant (ASV). All ASVs with solid points were significant after Benjamini–Hochberg multiple testing correction. The colored bar next to the taxonomic name of the ASV indicates the phylogenetic family. A numeric suffix was appended to features with identical taxonomic classifications to ensure unique names.

The age-adjusted Cox regression models with gut microbes as predictors and MACE as outcome resulted in 37 ASVs, of which three ASVs remained significant after multiple testing correction (Fig. 3A**;** Supplementary Table 3). *Lachnospiraceae* spp. (HR 0.84 (95% CI 0.77, 0.93) per CLR increase, *q* = 0.035, age-adjusted model) and *Akkermansia muciniphila* (HR 0.86 (95%CI 0.80, 0.94), *q* = 0.035) were associated with lower MACE incidence, while *Allisonella histaminiformans* (HR 1.24 (95% CI 1.10, 1.39), *q* = 0.035) was associated with increased MACE incidence. These associations lost significance after adjusting for sex, BMI, smoking, and alcohol use.

We performed similar analyses with MACE+ as outcome (Fig. 3B**;** Supplementary Table 4). These Cox regression models resulted in 62 ASVs that were associated with MACE+, of which 22 ASVs were significant after multiple testing correction. While one ASV, *Ruminococcus gnavus group* spp., was associated with a higher incidence of MACE+ (HR 1.10 (95%CI 1.03–1.18), *q* = 0.040) in the age-adjusted model, all other ASVs were associated with a lower incidence. Two MACE-predicting ASVs were also associated with MACE+: *Lachnospiraceae* spp. (HR 0.87 (95% CI 0.80–0.94), *q* = 0.031) and *Akkermansia muciniphila* (HR 0.90 (95% CI 0.84–0.96), *q* = 0.031). Other protective ASVs with the strongest effect sizes were *Eubacterium xylanophilum group* spp. (HR 0.81 (95% CI 0.74–0.88), *q* < 0.001) and *Lachnospiraceae GCA-900066575* spp. (HR 0.82 (95% CI 0.72–0.92), *q* = 0.031). The association with *Eubacterium xylanophilum group* spp. was the only ASV that remained significant after adjustment for sex, BMI, smoking, and alcohol use (HR 0.85 (95% CI 0.77–0.92), *q* = 0.039).

We also stratified the associations for the three largest ethnic groups, Dutch, African Surinamese, and South-Asian Surinamese, to explore whether the associations were different between ethnicities (Supplementary Fig. 2). We did not find any significant interactions with ethnicity across the 23 associations with MACE and MACE+. The associations between gut microbiota and MACE seemed to be predominantly driven by Dutch and African Surinamese participants, while they were often absent in South-Asian Surinamese participants. Sex stratification showed that associations were stronger in men than women (e.g., for *Eubacterium xylanophilum group* spp. and *Ruminococcus gnavus group* spp.; Supplementary Fig. 3). Likely, this was caused by lower power in the latter subgroups (South-Asian Surinamese and women), since the associations showed much fewer differences between ethnicities and sexes in the MACE+ associations with a higher number of events. Next, we investigated associations between dietary variables (macronutrient groups and sodium intake) and MACE to assess if these could serve as confounders for the associations between gut microbiota and MACE; however, none of these variables showed significant associations with MACE (Supplementary Table 5). Lastly, to take into account the competing risk of non-cardiovascular death, we additionally performed Fine-Gray analyses to estimate the subdistribution hazard ratios (sHR) for the ASVs significant in the previous analyses, while taking into account the competing risk of non-cardiovascular death (Supplementary Fig. 4). The associations with MACE and MACE+ showed minimal changes compared to the Cox regression models, hence competing risk seemed to be of limited importance.

### Gut microbiota composition and new cardiometabolic diagnoses at follow-up

**Fig. 4:**
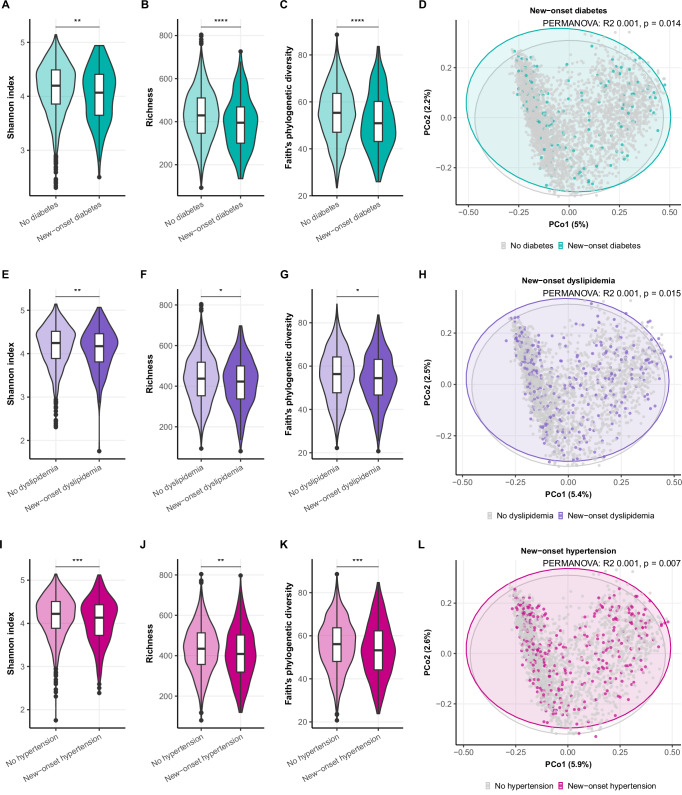
Microbial diversity and new-onset cardiometabolic diagnoses. New-onset diabetes (**A**–**D**), dyslipidemia (**E**–**H**), and hypertension (**I**–**L**), and baseline diversity in the gut microbiome of 3511 participants. Alpha diversity indices, including Shannon index (**A**, **E**, **I**), richness (**B**, **F**, **J**), and Faith’s phylogenetic diversity (**C**, **G**, **K**); differences tested with Mann–Whitney U tests. Beta diversity as measured with Bray–Curtis distances is shown in principle coordinate analyses plots, differences tested with permuted analysis of variance (PERMANOVA). *p < 0.05, **p < 0.01, ***p < 0.001.

Next, we explored associations between baseline gut microbiota composition and new cardiometabolic diagnoses at follow-up visit, including hypertension, dyslipidemia, and diabetes. First, we looked at baseline differences in alpha and beta diversity between participants with and without the new-onset diagnoses of interest at follow-up (Fig. 4). Participants with new-onset diabetes, dyslipidemia, or hypertension at follow-up had significantly lower alpha diversity at baseline, reflected by the Shannon index, richness, and Faith’s phylogenetic diversity. However, the absolute differences between groups were small. Beta diversity, as measured with Bray-Curtis distances, also showed minor yet significant differences between groups with and without new-onset diagnoses.

**Fig. 5:**
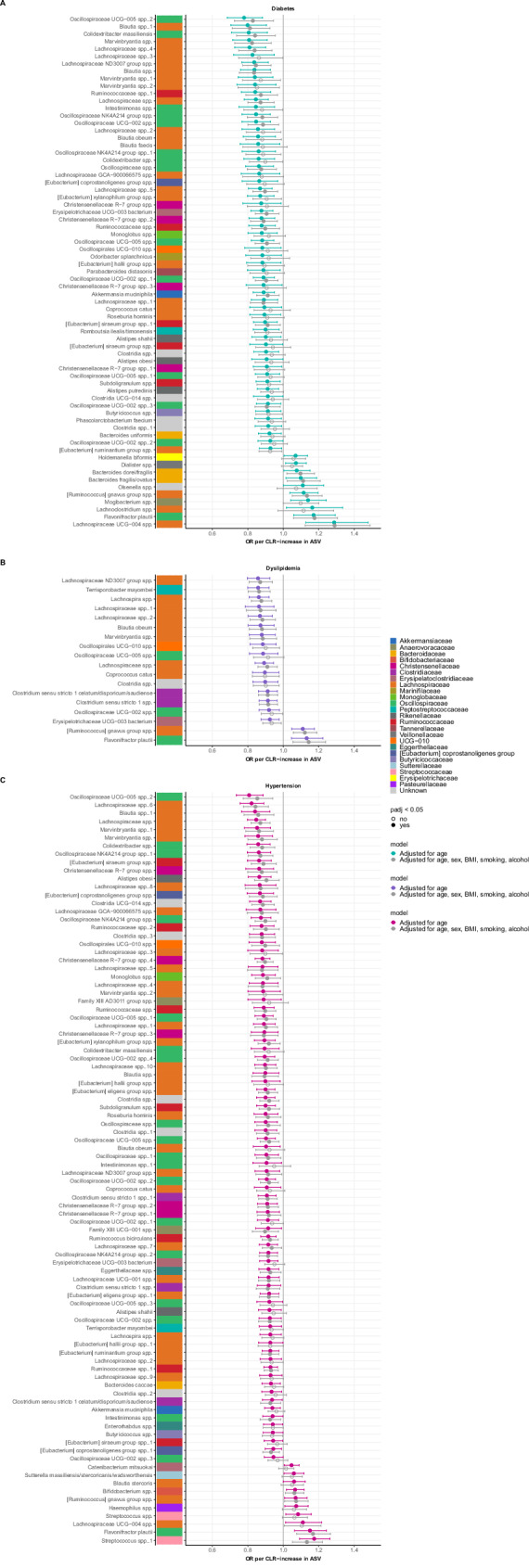
Logistic regression models: baseline gut microbes and new-onset cardiometabolic diagnoses. Age-adjusted regression models per outcome: diabetes (**A**), dyslipidemia (**B**), and hypertension (**C**) in 3511 participants. Forest plots with estimates (odds ratios) and 95% confidence intervals per centered log-ratio increase in the amplicon sequence variant (ASV). All ASVs shown were significant after Benjamini–Hochberg multiple testing correction. The colored bar next to the taxonomic name of the ASV indicates the phylogenetic family. A numeric suffix was appended to features with identical taxonomic classifications to ensure unique names.

We used logistic regression with 225 microbes (ASVs) as determinants and occurrence of new cardiometabolic diagnoses at follow-up as outcomes. The age-adjusted regression models revealed 69 microbes that were associated with new-onset diabetes after multiple testing correction (Fig. 5A; Supplementary Table 6), of which 27 remained significant after adjusting for sex, BMI, smoking, and alcohol use. Most of these ASVs were associated with lower odds of new-onset diabetes, while 5 ASVs were associated with higher odds of diabetes at the follow-up visit. These ASVs included *Lachnospiraceae UCG-004* spp. (OR 1.29 (95% CI 1.12–1.49) per CLR increase, *q* = 0.016; fully adjusted model), *Flavonifractor plautii* (OR 1.18 (95% CI 1.06–1.31), *q* = 0.019), *Ruminococcus gnavus group* spp. (OR 1.13 (95% CI 1.05–1.22), *q* = 0.016).

In the models for new-onset dyslipidemia (Fig. 5B; Supplemental Table 7), 18 microbes showed associations in the age-adjusted models, of which 12 were still significant in the fully adjusted model. Two ASVs were associated with higher odds of dyslipidemia: *Ruminococcus gnavus group* spp. (OR 1.12 (95% CI 1.06–1.19), *q* = 0.004) and *Flavonifractor plautii* (OR 1.14 (95% CI 1.05–1.24), *q* = 0.020). The other 9 ASVs were associated with lower odds of dyslipidemia, most of which belonged to the *Lachnospiraceae* family (7 ASVs).

**Fig. 6:**
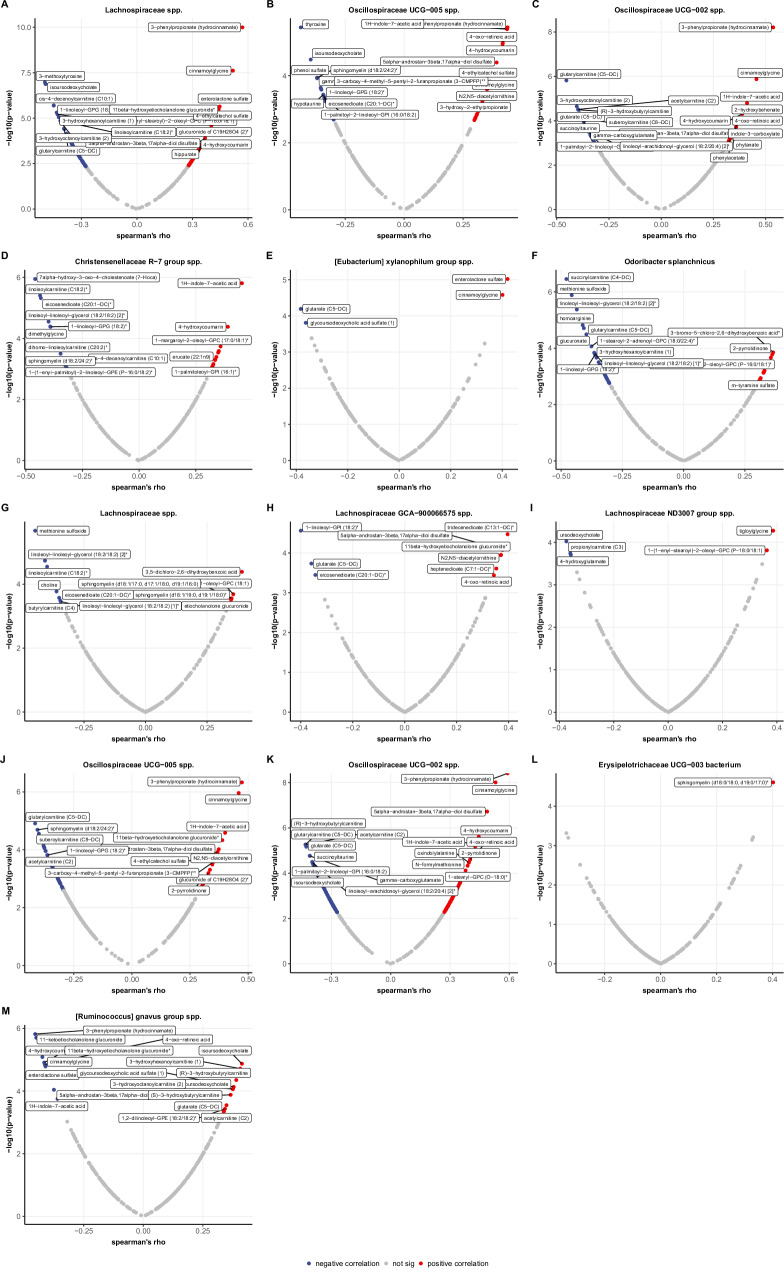
Volcano plots of correlations between MACE-associated microbes and serum metabolites. Plots with Spearman’s correlation coefficients (rho) and signifi cance level per MACE-predicting ASV (A-M) in 105participants, showing all correlations between the annotated serum metabolites and the abundance of the ASV. All colored points are correlations that were significant after multiple testing correction.

With regards to the incidence of hypertension, we found associations with 92 ASVs, of which 62 were still significant after full adjustment (Fig. 6C; Supplementary Table 8). Four ASVs were associated with higher odds of hypertension, while 58 ASVs were associated with lower odds of hypertension. The four ASVs that were associated with new-onset hypertension included *Streptococcus* spp. (OR 1.13 (1.05–1.22), *q* = 0.010; fully adjusted model), *Flavonifractor plautii* (OR 1.17 (1.08–1.27), *q* = 0.004), *Ruminococcus gnavus group* spp. (OR 1.07 (1.01–1.14), *q* = 0.043) and *Bifidobacterium* spp. (OR 1.06 (1.01–1.12), *q* = 0.027). Several 20 ASVs from the *Lachnospiraceae* family, including *Blautia, Marvinbryantia,* and *Eubacterium*, that were associated with lower odds of hypertension. Additionally, there were 13 *Oscillosporaceae* ASVs, including *Colidextribacter*, 6 *Ruminococcaceae* ASVs, such as *Ruminococcus bicirculans*, and 5 *Christensenellaceae* ASVs associated with lower hypertension incidence over the 6 years of follow-up.

We assessed if there were interactions with ethnicity across the associations between gut microbes and the observed cardiometabolic diagnoses in the three largest ethnic groups: Dutch, South-Asian Surinamese, and African Surinamese (Supplementary Fig. 5). In the analyses for new-onset diabetes, three ASVs (*Eubacterium coprostaligenes group* spp., *Bacteroides fragilis/ovatus*, and *Mogibacterium* spp.) showed significant interactions with ethnicity, with stronger associations in participants of African Surinamese and South-Asian Surinamese origin compared with those of Dutch origin. Whereas only two ASVs associated with dyslipidemia showed significant ethnicity interactions, 13 hypertension-associated ASVs showed such interactions. These ASVs were primarily of the *Lachnospiraceae* and *Oscillosporaceae* phylogenetic families. Stratification showed that the (predominantly protective) associations with new-onset hypertension were stronger in participants of Dutch and African-Surinamese origin compared to those of South-Asian Surinamese origin. When stratified by sex (Supplementary Fig. 6), associations with new-onset diabetes—and to a lesser extent hypertension—appeared to be stronger in women than in men for both protective and disease-associated ASVs. For diabetes, this resulted in 11 significant sex interactions across five different phylogenetic families (*Oscillosporaceae*, *Lachnospiraceae*, *Christenellaceae,* and *Bacteroidaceae*).

### MACE-predicting gut microbes and serum metabolites

To identify metabolites correlated with the 23 ASVs derived from the MACE analyses (age-adjusted models), we performed correlation analyses using baseline serum metabolomics data in a subgroup of 105 HELIUS participants (Supplementary Table 9**;** Fig. 6). After Benjamini–Hochberg multiple testing correction, we found correlations between 13 ASVs and 218 serum metabolites.

Of these ASVs, only *Ruminococcus gnavus group* spp. was associated with higher MACE incidence. This ASV was correlated with 20 serum metabolites, and showed the strongest positive correlation with isoursodeoxycholate (rho 0.41, *q* = 0.002; Supplementary Fig. 7), a secondary bile acid, followed by two carnitine metabolites, 3-hydroxyhexanoylcarnitine and (R)-3-hydroxybutyrylcarnitine (rho 0.39–0.41). In contrast, *Ruminococcus gnavus group* spp. was negatively correlated with 3-phenylpropionate (also known as hydrocinnamate; rho –0.45, *q* = 0.001), a xenobiotic metabolite, and 11-ketoetiocholanolone glucuronide (rho –0.45, *q* = 0.001), an androgenic steroid.

The other 12 ASVs were associated with lower odds of MACE. These ASVs showed the strongest positive correlations with xenobiotic metabolites, including 3-phenylpropionate (rho between 0.35 and 0.59), followed by cinnamoylglycine (rho 0.35–0.53) and the microbial-derived enterolactone sulfate (rho 0.33–0.45). Cinnamoylglycine is a product of the microbial metabolization of cinnamic acid (found in fruits, vegetables, and spices, e.g., cinnamon), while enterolactone sulfate is a microbial product of dietary lignans (present in seeds and whole grains). Another bacterial metabolite that showed positive correlations with 6 ASVs was 1H-indole-7-acetic acid (rho 0.34–0.45). Interestingly, the androgenic steroid 5alpha-androstan-3beta,17alpha-diol disulfate was also positively correlated with 7 ASVs (rho 0.35–0.49). Additionally, we found 4-hydroxycoumarin to be correlated with 6 ASVs (rho 0.31–0.45), which could be a metabolite of coumarin anticoagulants, a microbial product, or a dietary plant-based metabolite^12,13^. Some metabolites were negatively correlated with these ASVs, including 3-methoxytyrosine, a tyrosine pathway metabolite that correlated with 3 ASVs (rho between −0.30 and −0.50), and isoursodeoxycholate, a secondary bile acid that correlated negatively with the abundance of 5 ASVs (rho between −0.32 and −0.49).

## Discussion

We investigated the long-term associations between gut microbiota composition and cardiometabolic health in the multi-ethnic HELIUS cohort. Our findings underscore the associations between gut microbiota composition and cardiometabolic outcomes, including new-onset cardiometabolic diagnoses and MACE. *Allisonella histaminiformans* and *Ruminococcus gnavus group* spp. were associated with a higher incidence of cardiovascular events, although these associations were no longer significant after adjustment for other risk factors. Additionally, we identified a number of microbes associated with new-onset cardiometabolic diagnoses after a 6-year follow-up, including *Flavonifractor plautii*, *Streptococcus* spp., and *Bifidobacterium* spp.

In age-adjusted analyses, we found *Lachnospiraceae* spp. and *Akkermansia muciniphila* to be protective, while *Allisonella histaminiformans* and *Ruminococcus gnavus group* spp. were MACE-associated. *Allisonella histaminiformans* is, as the name suggests, a histamine-producing microbe. Systemic histamine has been associated with atherogenesis in experimental models^14–16^. *Ruminococcus gnavus group* spp. was correlated with semi-quantitative levels of a secondary bile acid and acylcarnitines. Secondary bile acid can modulate hepatic lipid and cholesterol metabolism via FXR and TGR5 signaling and influence atherogenesis, although they are generally considered cardiometabolically protective^17,18^. Medium-chain acylcarnitines are β-oxidation intermediates, and their accumulation may reflect impaired fatty acid oxidation, which has been associated with a higher risk of cardiovascular disease and diabetes^19–21^.

Adjusting for confounders, including sex, BMI, smoking, and alcohol, attenuated the associations between gut microbes and MACE/MACE + . This illustrates that the gut microbiome reflects lifestyle and cardiometabolic risk factors, serving as an early indicator of cardiovascular risk that cannot be seen outside of the context of other lifestyle factors. However, the effect of some confounders, such as BMI, is difficult to interpret, as gut microbes may influence energy harvest and weight gain^22^, placing BMI potentially on the causal pathway to cardiovascular events.

Although diet shapes gut microbiome composition, we did not observe associations between nutrient groups and MACE, precluding their inclusion as confounders in these analyses. However, our serum metabolomics analyses revealed correlations of protective ASVs with xenobiotic and plant-derived metabolites, illustrating potential diet-microbe interactions. These metabolites included 3-phenylpropionate, cinnamoylglycine, and enterolactone sulfate, reflecting microbial processing of dietary components such as fruits, vegetables, spices, and whole grains^23–25^. Several of these metabolites have been associated with higher microbial diversity and lower cardiovascular risk in other population-based studies^26–28^.

We extended our analyses beyond MACE incidence by including cardiometabolic diagnoses that impact vascular inflammation, enabling comparison of our findings with cross-sectional evidence on these conditions. Studies in patients with type 2 diabetes have reported lower abundances of the genera *Bifidobacterium*, *Bacteroides*, *Faecalibacterium*, *Akkermansia,* and *Roseburia*, and higher *Ruminococcus gnavus* spp. and *Flavonifractor plautii* abundance^29–33^. In dyslipidemia, *Ruminococcus gnavus* spp., *Alistipes shahii*, and *Lachnospira eligens* have been cross-sectionally shown to be associated, while *Streptococcus*, *Klebsiella,* and *Parabacteroides* spp. have been associated with higher BP and hypertension^6,34–41^. In our longitudinal analyses, several previously reported associations were confirmed. Protective associations were observed for *Blautia*, *Marvinbryantia*, and *Akkermansia*, whereas *Streptococcus*, *Flavonifractor plautii*, and *Ruminococcus gnavus* were associated with increased cardiometabolic disease incidence. Comparing with existing longitudinal studies on type 2 diabetes, we could not replicate associations with *Butyrivibrio* and *Faecalibacterium* reported by Wang et al., or *Clostridium citronae* and *Enterocloster bolteae* reported by Ruuskanen et al.^26,42^. This may relate to differences in cohort characteristics and analyses approaches.

Interestingly, we found *Bifidobacterium* to be associated with a higher incidence of hypertension, contrasting with the generally positive effects described in the literature^43–45^, although a cross-sectional association between *Bifidobacterium adolescentis* and 24-h blood pressure levels has been reported in the Swedish SCAPIS study^46^. Recent findings suggest that *Bifidobacterium* spp. increase in abundance during urbanization and associate with cardiovascular risk factors, indicating potentially heterogenic effects among species within this genus^47^.

Sex-stratified analyses revealed substantially larger effect sizes in women for associations with new-onset diabetes, and to a lesser extent, hypertension, for both protective and disease-associated microbes. These findings align with previous studies showing that premenopausal women often harbor higher abundances of taxa with beneficial metabolic effects^48^ and with previous results of the HELIUS study^49^, which demonstrated modest but consistent associations between sex and gut microbial composition. The stronger associations we observed in women may reflect sex-specific metabolic responses to gut microbes. Evidence from mouse models suggests that sex differences in glucose metabolism depend on gut microbiome-hormone interactions, as depletion of the microbiome abolishes sex differences in glucose regulation and insulin sensitivity^50^. Together, these observations support the use of sex-stratified approaches in future studies.

The multi-ethnic nature of the HELIUS cohort is one of the strengths of this study, since it improves the generalizability of our findings. The availability of follow-up visit data with consistent methods across both timepoints allowed us to investigate changes in cardiometabolic diagnoses affecting atherosclerosis, while registry analyses enabled us to investigate the associations with cardiovascular endpoints. This dual approach offered a comprehensive view of cardiometabolic disease in this population, spanning earlier risk factors to clinical outcomes.

Limitations of our study include the following. The fecal microbiota composition measurement was based on a single baseline fecal sample, which may not capture microbiome variability over time^51^. Our analyses might be affected by selection and attrition bias and inaccurate reporting of medication use. Non-response analyses in the HELIUS cohort revealed that participants who took part in the first follow-up were slightly healthier than those who did not^52^, as was also reflected by our comparisons in the microbiome subcohort. The incidence of cardiometabolic diagnoses may, as a result, be an underestimation of the true rates, but we do not expect that this has influenced the associations between gut microbes and diagnoses. In contrast, the registry analyses were not affected by loss to follow-up. Registry data rely on accurate reporting, which could be complicated by longer stays abroad, potentially leading to underreporting in migrant populations. However, a Danish study on remigration bias in mortality analyses could not confirm this hypothesis^53,54^. Finally, we were unable to externally validate our findings in an independent cohort, which should be addressed in future studies.

Our findings provide novel insights into the temporality of associations between gut microbes and cardiometabolic disease. However, it remains plausible that the gut microbiota reflects the cumulative impact of various lifelong risk factors rather than serving as an independent driver. While the gut microbiota may have limited utility as a diagnostic tool, it holds promise as a target for interventions aimed at preventing low-grade vascular inflammation long before cardiovascular events manifest. Our metabolite analyses highlighted interactions between gut microbes and plant-based dietary components, emphasizing the potential benefits of plant-based diets in modulating microbiota-associated cardiovascular risk^55^. Alternative approaches, such as fecal microbiota transplantation, though effective, require frequent repetition (approximately every 2 months) and are thus impractical for long-term management. The supplementation of specific microbial strains tailored to support cardiovascular health may represent a more sustainable strategy, although evidence for their effectiveness remains limited, and further research is needed.

Our study shows that gut microbiota are longitudinally associated with cardiometabolic outcomes—including MACE, diabetes, dyslipidemia, and hypertension—in a cardiovascular disease-naïve population. Metabolomics analyses identified key metabolites that may mediate the effects of the disease-associated *Ruminococcus gnavus group* spp. and the potential protective effects of *Lachnospiraceae* and *Oscillosporaceae* microbes. These findings provide a foundation for future research to develop microbiome-targeted strategies for reducing cardiovascular risk and improving cardiometabolic health.

## Methods

### Study population

The Healthy Life in an Urban Setting (HELIUS) study is a prospective multi-ethnic population-based cohort study (*N* = 24,780) conducted in Amsterdam, the Netherlands, with baseline visits between 2011 and 2015^56^. Individuals between 18 and 70 years of age were randomly sampled from the municipality registry while stratifying for ethnicity (Dutch, Surinamese, Ghanaian, Turkish, or Moroccan origin) to ensure representation across ethnic groups. All baseline participants were invited for follow-up visits between 2019 and 2022, of which 11,035 participated (44.5%). Informed consent was obtained from all participants, the study was approved by the medical ethical review board (Amsterdam UMC, location AMC), and followed the principles of the Declaration of Helsinki.

Participants were defined as of non-Dutch ethnic origin if they were born outside the Netherlands and at least one parent was born abroad (first generation), or if they were born in the Netherlands but both parents were born abroad (second generation). For the Dutch group, we invited participants that were born in the Netherlands and whose parents were born in the Netherlands. Participants of Surinamese ethnic origin were further categorized according to self-reported ethnic origin into (e.g., “African” or “South-Asian”).

Prior to the study visits, participants were instructed to abstain from smoking. Office blood pressure (BP) was measured after 5 min of rest in the supine position, using the average of two readings from a validated semi-automatic oscillometric device (Microlife WatchBP Home; Microlife AG, Switzerland). Venous blood samples were obtained after a fasting period of at least 10 h, and serum samples were stored at −80 °C until further analysis. Hypertension was defined as either an elevated BP (systolic BP equal or higher than 140 mmHg or diastolic blood pressure equal or higher than 90 mmHg), or use of antihypertensive medication. Diabetes was defined as the use of glucose-lowering drugs or elevated glucose levels (7.0 mmol/L or higher). Dyslipidemia was defined as triglycerides ≥1.7 mmol/L, and high-density lipoprotein cholesterol <1.0 mmol, or the use of lipid-lowering drugs^57^. History of cardiovascular disease was defined as a self-reported history of stroke, myocardial infarction, or revascularization. Dietary intake information was collected using food frequency questionnaires^58^, from which daily average intake of energy (kcal), macronutrient groups (carbohydrates, fat, fibers, proteins, and alcohol intake; all in grams), and sodium intake (in grams) was derived.

For the current analyses, we included all participants with available fecal samples at baseline that gave consent for linkage of their data with hospital and mortality registries (*N* = 5160). Of these participants, 3533 individuals also participated in a follow-up visit between 2019 and 2022 (60.2%). In reporting our analyses, we followed the STROBE guidelines; the STROBE checklist can be found in Supplementary Table 10^59^.

### Cardiovascular outcomes

Primary outcomes for our analyses were MACE. These data were derived from hospital (Dutch Hospital Data) and mortality registries from January 1st, 2011, up to January 1st, 2024, which are non-public microdata owned by Statistics Netherlands. For these analyses, we included all participants with gut microbiome baseline data that gave permission for linking their data to the population registry, and in whom linkage succeeded (*N* = 5122). We used International Classification of Diseases 10th Revision (ICD) codes from these registries to capture 4-point MACE-4. This outcome included cardiovascular death, hospitalizations for myocardial infarctions and stroke, and coronary revascularization, following the definitions used for risk estimation in SCORE-2^60^. Supplementary Table 11 provides an overview of included and excluded ICD codes. For participants experiencing multiple MACE-4 events during follow-up, the time to the first event was used. We also used a broader cardiovascular adverse event outcome (MACE+) for our analyses, that additionally included hospitalizations for angina pectoris, to reflect underlying atherosclerotic vascular pathology and increase statistical power. Non-cardiovascular death was defined as mortality that was not caused by MACE-4 according to the ICD codes, and was regarded as a competing risk.

Secondary cardiovascular outcomes included the incidence of new cardiometabolic diagnoses between baseline and follow-up, defined as a diagnosis of hypertension, dyslipidemia, or diabetes at follow-up that was absent at baseline. The diagnosis definitions as described above (“Study population”) were used both at baseline and follow-up. Since these diagnoses were established at the follow-up visit, we did not have exact data on the time of diagnosis. The advantage of using these data over hospital registry data is that it provides a better estimate of the occurrence of diagnoses for which no hospital admission is indicated. These outcomes could be defined in a group of 3108 participants with both baseline and follow-up data.

### Gut microbiota composition

Gut microbiota composition was determined from baseline fecal samples (*N* = 6028; see Fig. 1 for a flowchart of the selection of participants). All HELIUS participants were asked to bring a fresh fecal sample within 6 h after collection, or, if not possible, to store the sample overnight in a freezer. Samples were stored at −20 °C at the study visit location for a maximum of 1 day before transportation to the central freezer (−80 °C). Samples were shipped to the Wallenberg Laboratory (Sahlgrenska Academy at University of Gothenburg, Sweden) for sequencing. DNA was extracted from a 150 mg aliquot of fecal samples using a repeated bead-beating protocol^61^. Fecal microbiota composition was determined by sequencing the V4 region of the 16S rRNA gene on an Illumina MiSeq (Illumina RTA v1.17.28; MCS v2.5, San Diego, CA, USA) using 515 F and 806 R primers designed for dual-indexing and the V2 Illumina kit (2 × 250 bp paired-end reads)^62^. PCR was performed in duplicate reactions as previously described^5^.

Sequencing reads were processed using a publicly shared Nextflow workflow to infer amplicon sequence variants (ASVs)^63^. Using VSEARCH (v.2.27.0), fastqs were merged (*fastq_mergepairs*) and quality filtered (*fastq_filter*), reads were dereplicated (*fastx_uniques*), denoising was performed with alpha 2 and minimum size of 8 to infer ASVs (*cluster_unoise*), and singletons (*sortbysize*) and chimeras (*uchime_denovo3*) were removed. Finally, reads were mapped to the ASVs (*usearch_global*, *--id 0.97*)^64^. Taxonomy was assigned with the *assignTaxonomy* function of the DADA2 package using the SILVA database (v.138.1). The count table was rarefied to 15,000 counts per sample, resulting in the exclusion of 14 samples due to insufficient counts. Rarefaction curves indicated that richness stabilized well below the rarefaction depth of 15,000 reads (Supplementary Fig. 1). Next, we removed samples of participants that used antibiotics within 3 months prior to sample collection. The workflow resulted in two datasets: one dataset of individuals with paired clinical data (baseline and follow-up visits) comprising 3511 participants and 7636 taxa, and one dataset of individuals with baseline HELIUS data (baseline visit and registry data) comprising 4792 samples and 7783 taxa.

### Serum metabolomics

The metabolomics subcohort was selected by matching participants of Dutch and South-Asian Surinamese ethnicity on age, sex, and BMI using the MatchIt R package (nearest-neighbor matching; Supplementary Table 3). Baseline serum samples (*N* = 105) were sent to Metabolon (Morrisville, North Carolina, USA) for untargeted metabolite profiling using ultra-high-performance liquid chromatography coupled to tandem mass spectrometry (UPLC-MS/MS). All analyses operated at a mass resolution of 35,000, identifying compounds by comparing them to library entries. Missing values were assumed to be a consequence of concentrations being lower than detection limits, and were therefore imputed with the minimal observed value. We excluded all metabolites that were not annotated and log10-transformed all (zero-mean-unit-variance normalized) metabolite abundances. The resulting dataset comprised 1148 metabolites.

### Statistics

Baseline characteristics were presented for all included participants with registry data (MACE analyses) and for participants with baseline and follow-up data (new-onset diagnoses analyses). Baseline characteristics of participants lost to follow-up were compared with those retained to assess potential selection bias. We calculated alpha diversity metrics (Shannon index, richness, and Faith’s phylogenetic diversity) and Bray–Curtis distances using the vegan and picante packages. Alpha diversity group differences were tested with Mann–Whitney U tests; beta diversity differences were tested with PERMANOVA with 999 permutations. We used age-adjusted Cox regressions to analyze associations between gut microbial abundances and MACE or MACE-plus, while excluding participants with self-reported cardiovascular disease or MACE events occurring prior to their baseline visit (with available data from 2011). We filtered the ASV table for ASVs with 5 counts in at least 20% of subjects (222 ASVs for the MACE analyses; 225 ASVs for the new-onset diagnoses analyses). Prior to the analyses, we centred log-ratio (CLR)-transformed all ASVs after adding 0.5 pseudocount. In the first model, we included baseline age as a covariate, while in the second model, we additionally included sex, BMI, smoking, and alcohol use. In subgroup analyses, we stratified age-adjusted models for the three largest ethnic groups, Dutch, African Surinamese, and South-Asian Surinamese, and tested for the ASV*ethnicity interaction in the age-adjusted unstratified models with these three ethnicities. We performed identical models with sex-stratification and testing for interactions with sex. We then performed Fine-Gray models focusing on the MACE-associated ASVs of the previous analyses to take into account the competing risk of non-cardiovascular death. There were no missing data in these analyses.

To investigate the associations between baseline gut microbiota composition and new diagnosis at follow-up, we first excluded all participants with the diagnosis of interest at baseline, separately for each diagnosis. We performed logistic regression models with the ASV abundances (filtered and transformed as described above) as determinants and new diagnoses (binary; separate models for diabetes, dyslipidemia, and hypertension) as outcomes. In the first model, we included baseline age as covariate, while in the second model, we additionally included sex, BMI, smoking, and alcohol use. Data on incident diabetes, dyslipidemia, and hypertension were missing for 27 participants (0.8%), 11 participants (0.3%), and 3 participants (0.09%), respectively. These participants were excluded specifically for the analyses using these outcomes. As in the MACE analyses, we repeated all age-adjusted models in subgroups for ethnicity and sex and tested for interactions in the unstratified model (ASV*ethnicity and ASV*sex interaction terms).

Lastly, we used Spearman’s correlations between the MACE-predicting microbes (significant in age-adjusted models) and serum metabolite levels to explore underlying mechanisms. We performed Spearman’s correlations between microbe abundances (CLR-transformed) and metabolite concentrations (log10-transformed).

All *p*-values were adjusted using the Benjamini–Hochberg method (reported as *q*-values) and considered significant when lower than 0.05. Significance levels of interaction testing were set at *p* < 0.05, without multiple testing adjustment. Statistical analyses and visualizations were performed using R version 4.4.3 for the MACE analyses and visualizations and in R version 4.5.1 for all other analyses.

## Supplementary information

Supplementary Information

## Data Availability

The Amsterdam UMC, located at AMC in Amsterdam, The Netherlands, owns the HELIUS data. Researchers interested in accessing this data can submit proposals to the HELIUS Executive Board via http://www.heliusstudy.nl/en/researchers/collaboration. The HELIUS Executive Board will evaluate proposals for alignment with the study’s overall objectives, ethical approvals, and informed consent forms. The 16S microbiota sequencing data have been deposited in a repository of the European Genome-Phenome Archive (https://ega-archive.org/studies/EGAS00001002969). The population and hospital registry data of Statistics Netherlands (Centraal Bureau voor de Statistiek; CBS) are accessible for statistical and scientific research under certain conditions. For further information, please contact the CBS Microdata department at microdata@cbs.nl.

## Code availability

The programming code used for the analyses was publicly shared in a Github repository (https://github.com/barbarahelena/helius_cardiovascular).
