# Supplementary material for: Specific gut microbes are associated with the incidence of cardiometabolic disease in the HELIUS cohort
Source: NPJ Biofilms Microbiomes. 2026 Mar 7;12:83. doi: 10.1038/s41522-026-00952-6 (PMC13096484; doi:10.1038/s41522-026-00952-6)
Supplement: Supplementary file 1 — Supplementary Information [file 41522_2026_952_MOESM1_ESM.pdf]

SUPPLEMENTS: Specific gut microbes are associated with the incidence of cardiometabolic disease in the HELIUS cohort

Barbara J.H. Verhaar,[1,2,3] Thomas A. Bouwmeester,[1,2] Henrike Galenkamp,[4,5] Bert-Jan H. van den Born,[1,2,4] Max Nieuwdorp,[1,6]

1 Department of Vascular Medicine, Amsterdam UMC, Amsterdam, The Netherlands

2 Amsterdam Cardiovascular Sciences, Amsterdam UMC, University of Amsterdam, Amsterdam, The Netherlands

3 Amsterdam Gastroenterology Endocrinology Metabolism, Amsterdam UMC, University of Amsterdam, Amsterdam, The Netherlands

4 Department of Public and Occupational Health, Amsterdam UMC, location AMC, Amsterdam, The Netherlands

5 Amsterdam Public Health Research Institute, Amsterdam UMC, Amsterdam, The Netherlands

6 Department of Experimental Vascular Medicine, Amsterdam UMC, Amsterdam, The Netherlands

Corresponding author: Barbara Verhaar, b.j.verhaar@amsterdamumc.nl

Supplementary Table 1: Baseline population characteristics stratified for ethnicity

A: Subset with registry data

	**European Dutch**	**South-Asian Surinamese**	**African Surinamese**
**n**	1433	643	1160
**Age (years)**	51.1±12.8	51.1±11.2	51.8±10.6
**Women**	719 (50.2)	357 (55.5)	672 (57.9)
**BMI (kg/m^2)**	25.4±4.3	26.6±4.4	28.2±5.4
**Smoking status**			
Yes	285 (19.9)	139 (21.8)	312 (27.1)
Never	533 (37.2)	393 (61.6)	563 (48.8)
Former smoking	613 (42.8)	106 (16.6)	278 (24.1)
**Diabetes**	66 (4.6)	143 (22.3)	149 (12.9)
**Systolic BP (mmHg)**	127.0±17.2	131.1±19.2	133.7±18.2
**Diastolic BP (mmHg)**	79.2±10.3	81.3±10.3	83.6±10.5
**Hypertension**	447 (31.2)	298 (46.3)	622 (53.8)
**Dyslipidemia**	297 (20.7)	227 (35.3)	227 (19.6)
**Total cholesterol (mmol/L)**	5.3±1.0	5.0±1.1	5.0±1.0
**LDL cholesterol (mmol/L)**	3.3±0.9	3.1±0.9	3.1±0.9
**Triglycerides (mmol/L)**	0.9 [0.6, 1.3]	1.0 [0.7, 1.5]	0.8 [0.6, 1.1]
**HbA1c (mmol/mol)**	36.7±4.7	43.7±11.2	40.6±8.7
**Non-cardiovascular deaths**	49 (84.5)	20 (74.1)	55 (88.7)
**MACE4 events**	38 (2.6)	31 (4.8)	30 (2.6)
**MACE-plus event**	48 (3.3)	53 (8.2)	35 (3.0)

B: Subset with follow-up visits

	**European Dutch**	**South-Asian Surinamese**	**African Surinamese**
**n**	1149	530	863
**Age (years)**	52.2±11.8	51.9±10.3	52.9±9.7
**Women**	566 (49.3)	286 (54.0)	535 (62.0)
**BMI (kg/m^2)**	25.3±4.1	26.5±4.4	28.1±5.2
**Smoking status**			
Yes	209 (18.2)	105 (20.0)	196 (22.9)
Never	414 (36.1)	333 (63.3)	444 (51.9)
Former smoking	525 (45.7)	88 (16.7)	216 (25.2)
**Diabetes**	51 (4.5)	112 (21.2)	91 (10.6)
**Systolic BP (mmHg)**	126.5±16.6	130.5±17.4	133.1±17.9
**Diastolic BP (mmHg)**	78.9±9.9	81.3±9.6	83.0±10.3
**Hypertension**	368 (32.1)	235 (44.3)	468 (54.3)
**Dyslipidemia**	315 (27.5)	347 (54.1)	423 (36.6)
**Total cholesterol (mmol/L)**	5.3±1.0	5.0±1.1	5.0±1.0

**LDL cholesterol (mmol/L)**	3.3±0.9	3.1±0.9	3.1±0.9
**Triglycerides (mmol/L)**	0.9 [0.6, 1.3]	1.0 [0.8, 1.5]	0.8 [0.6, 1.1]
**HbA1c (mmol/mol)**	36.9±4.8	43.5±10.2	40.5±7.6

Data is presented as mean±SD, median [interquartile range] or n (%). BMI = body mass index, BP = blood pressure, LDL = low-density lipoprotein, MACE = major adverse cardiovascular event, MACE+ = major adverse cardiovascular event plus angina pectoris.

Supplementary Table 2: Baseline characteristics of population with and without follow-up data

	**Lost to follow-up**	**With follow-up visit**	**p**
**n**	2517	3511	
**Age (years)**	48.2 (12.6)	51.0 (10.8)	<0.001
**Women**	1340 (53.2)	1863 (53.1)	0.913
**BMI (kg/m2)**	28.1 (5.2)	26.9 (4.7)	<0.001
**Smoking**			<0.001
Yes	577 (24.2)	628 (18.0)	
Never	1278 (53.7)	1844 (52.8)	
Former smoking	525 (22.1)	1019 (29.2)	
**Diabetes (%)**	379 (15.1)	350 (10.0)	<0.001
**Systolic blood pressure (mmHg)**	130.7 (19.0)	129.1 (17.6)	0.001
**Diastolic blood pressure (mmHg)**	81.4 (11.1)	80.6 (10.2)	0.003
**Hypertension**	1102 (43.9)	1412 (40.2)	0.005
**Dyslipidemia**	1058 (42.2)	1192 (34.0)	<0.001
**Total cholesterol (mmol/L)**	4.9 (1.0)	5.1 (1.0)	<0.001
**LDL cholesterol (mmol/L)**	3.0 (0.9)	3.2 (0.9)	<0.001
**Triglycerides (mmol/L)**	0.9 [0.6, 1.3]	0.9 [0.6, 1.2]	0.043
**HbA1c (mmol/mol)**	40.3 (10.2)	39.3 (7.7)	<0.001

Data is presented as mean±SD, median [interquartile range] or n (%). BMI = body mass index, BP = blood pressure, LDL = low-density lipoprotein.

Supplementary Table 3: Cox regressions of gut microbiota and MACE

Tax	Model	HR	Lower CI	Higher CI	p-value	padj
Prevotella_9 copri	Adjusted for age	1.058	1.003	1.117	0.038	0.260
Oscillospiraceae UCG-005 spp..1	Adjusted for age	0.905	0.828	0.989	0.027	0.244
Lachnospiraceae spp.	Adjusted for age	0.842	0.766	0.925	0.000	0.035
Roseburia spp.	Adjusted for age	0.881	0.802	0.968	0.008	0.139
Roseburia hominis	Adjusted for age	0.861	0.772	0.959	0.007	0.139
Lachnospiraceae spp..3	Adjusted for age	0.900	0.811	0.999	0.047	0.287
Christensenellaceae R-7 group spp.	Adjusted for age	0.896	0.812	0.989	0.030	0.244
Firmicutes spp.	Adjusted for age	0.888	0.802	0.982	0.021	0.236
Bifidobacterium bifidum	Adjusted for age	1.090	1.003	1.184	0.042	0.269
Oscillospiraceae NK4A214 group spp.	Adjusted for age	0.892	0.801	0.993	0.036	0.257
Anaerostipes hadrus	Adjusted for age	0.864	0.774	0.965	0.009	0.144
Bilophila wadsworthia	Adjusted for age	0.876	0.790	0.970	0.011	0.162
Paraprevotella spp.	Adjusted for age	0.898	0.809	0.997	0.043	0.269
[Eubacterium] xylanophilum group spp.	Adjusted for age	0.844	0.763	0.933	0.001	0.052
Lachnospiraceae UCG-001 spp.	Adjusted for age	0.901	0.816	0.995	0.040	0.265
Lachnospira spp.	Adjusted for age	0.890	0.804	0.986	0.025	0.244
Lachnospiraceae spp..1	Adjusted for age	0.847	0.755	0.951	0.005	0.131
Alistipes shahii	Adjusted for age	0.877	0.789	0.976	0.016	0.205
Alistipes obesi	Adjusted for age	0.869	0.775	0.974	0.016	0.205
Odoribacter splanchnicus	Adjusted for age	0.865	0.760	0.984	0.028	0.244
Lachnospiraceae spp..2	Adjusted for age	0.883	0.790	0.988	0.030	0.244
Mogibacterium spp.	Adjusted for age	1.170	1.061	1.289	0.002	0.070
Parabacteroides distasonis	Adjusted for age	1.143	1.037	1.260	0.007	0.139
Colidextribacter spp.	Adjusted for age	0.841	0.745	0.950	0.005	0.133
Catenibacterium mitsuokai	Adjusted for age	1.084	1.021	1.151	0.008	0.139
Akkermansia muciniphila	Adjusted for age	0.863	0.795	0.937	0.000	0.035
Lachnospiraceae GCA-900066575 spp.	Adjusted for age	0.846	0.735	0.974	0.020	0.236
Lachnospiraceae ND3007 group spp.	Adjusted for age	0.899	0.814	0.992	0.034	0.257
Holdemanella spp.	Adjusted for age	1.080	1.011	1.154	0.022	0.243
Oscillospiraceae UCG-005 spp.	Adjusted for age	0.851	0.736	0.983	0.029	0.244
Allisonella histaminiformans	Adjusted for age	1.238	1.099	1.394	0.000	0.035
Oscillospiraceae UCG-002 spp.	Adjusted for age	0.926	0.857	1.000	0.049	0.288
Erysipelotrichaceae UCG-003 bacterium	Adjusted for age	0.888	0.821	0.961	0.003	0.097
[Eubacterium] eligens group spp.	Adjusted for age	0.881	0.811	0.957	0.003	0.097
[Eubacterium] eligens group spp..1	Adjusted for age	0.916	0.844	0.994	0.036	0.257
Prevotella_7 spp.	Adjusted for age	1.081	1.005	1.162	0.037	0.257

[Ruminococcus] gnavus group spp.	Adjusted for age	1.091	1.009	1.180	0.029	0.244
Prevotella_9 copri	Adjusted for age, sex, BMI, smoking and alcohol	1.028	0.974	1.085	0.312	0.775
Oscillospiraceae UCG-005 spp..1	Adjusted for age, sex, BMI, smoking and alcohol	0.928	0.848	1.016	0.106	0.758
Lachnospiraceae spp..1	Adjusted for age, sex, BMI, smoking and alcohol	0.894	0.812	0.985	0.024	0.585
Roseburia spp.	Adjusted for age, sex, BMI, smoking and alcohol	0.894	0.814	0.982	0.019	0.585
Roseburia hominis	Adjusted for age, sex, BMI, smoking and alcohol	0.909	0.813	1.016	0.093	0.758
Lachnospiraceae spp..3	Adjusted for age, sex, BMI, smoking and alcohol	0.916	0.824	1.019	0.106	0.758
Christensenellaceae R-7 group spp.	Adjusted for age, sex, BMI, smoking and alcohol	0.922	0.833	1.020	0.116	0.758
Firmicutes spp.	Adjusted for age, sex, BMI, smoking and alcohol	0.932	0.841	1.033	0.180	0.758
Bifidobacterium bifidum	Adjusted for age, sex, BMI, smoking and alcohol	1.082	0.995	1.177	0.065	0.697
Oscillospiraceae NK4A214 group spp.	Adjusted for age, sex, BMI, smoking and alcohol	0.932	0.835	1.041	0.211	0.758
Anaerostipes hadrus	Adjusted for age, sex, BMI, smoking and alcohol	0.898	0.802	1.005	0.061	0.697
Bilophila wadsworthia	Adjusted for age, sex, BMI, smoking and alcohol	0.908	0.819	1.007	0.067	0.697
Paraprevotella spp.	Adjusted for age, sex, BMI, smoking and alcohol	0.880	0.791	0.979	0.019	0.585
[Eubacterium] xylanophilum group spp.	Adjusted for age, sex, BMI, smoking and alcohol	0.893	0.807	0.989	0.030	0.585
Lachnospiraceae UCG-001 spp.	Adjusted for age, sex, BMI, smoking and alcohol	0.931	0.842	1.029	0.159	0.758
Lachnospira spp.	Adjusted for age, sex, BMI, smoking and alcohol	0.942	0.849	1.045	0.258	0.758
Lachnospiraceae spp.	Adjusted for age, sex, BMI, smoking and alcohol	0.866	0.771	0.973	0.016	0.585
Alistipes shahii	Adjusted for age, sex, BMI, smoking and alcohol	0.926	0.829	1.035	0.176	0.758
Alistipes obesi	Adjusted for age, sex, BMI, smoking and alcohol	0.921	0.817	1.037	0.172	0.758
Odoribacter splanchnicus	Adjusted for age, sex, BMI, smoking and alcohol	0.920	0.807	1.049	0.214	0.758
Lachnospiraceae spp..2	Adjusted for age, sex, BMI, smoking and alcohol	0.897	0.802	1.004	0.058	0.697
Mogibacterium spp.	Adjusted for age, sex, BMI, smoking and alcohol	1.116	1.011	1.231	0.029	0.585
Parabacteroides distasonis	Adjusted for age, sex, BMI, smoking and alcohol	1.143	1.035	1.261	0.008	0.585
Colidextribacter spp.	Adjusted for age, sex, BMI, smoking and alcohol	0.873	0.771	0.988	0.032	0.585
Catenibacterium mitsuokai	Adjusted for age, sex, BMI, smoking and alcohol	1.048	0.986	1.113	0.131	0.758
Akkermansia muciniphila	Adjusted for age, sex, BMI, smoking and alcohol	0.900	0.827	0.980	0.015	0.585
Lachnospiraceae GCA-900066575 spp.	Adjusted for age, sex, BMI, smoking and alcohol	0.862	0.747	0.995	0.043	0.665
Lachnospiraceae ND3007 group spp.	Adjusted for age, sex, BMI, smoking and alcohol	0.920	0.830	1.019	0.111	0.758
Holdemanella spp.	Adjusted for age, sex, BMI, smoking and alcohol	1.047	0.979	1.119	0.178	0.758
Oscillospiraceae UCG-005 spp.	Adjusted for age, sex, BMI, smoking and alcohol	0.889	0.767	1.029	0.116	0.758
Allisonella histaminiformans	Adjusted for age, sex, BMI, smoking and alcohol	1.139	1.008	1.286	0.036	0.608
Oscillospiraceae UCG-002 spp.	Adjusted for age, sex, BMI, smoking and alcohol	0.959	0.886	1.039	0.306	0.775

Erysipelotrichaceae UCG-003 bacterium	Adjusted for age, sex, BMI, smoking and alcohol	0.907	0.838	0.982	0.016	0.585
[Eubacterium] eligens group spp.	Adjusted for age, sex, BMI, smoking and alcohol	0.912	0.838	0.992	0.032	0.585
[Eubacterium] eligens group spp..1	Adjusted for age, sex, BMI, smoking and alcohol	0.952	0.874	1.037	0.257	0.758
Prevotella_7 spp.	Adjusted for age, sex, BMI, smoking and alcohol	1.048	0.974	1.127	0.213	0.758
[Ruminococcus] gnavus group spp.	Adjusted for age, sex, BMI, smoking and alcohol	1.076	0.995	1.165	0.067	0.697

Coefficients resulting from Cox regression models adjusted for age, including the hazard ratios (HR) and 95%-confidence intervals (CI). P-values were adjusted using the false discovery ratio. All amplicon sequence variants (ASVs) that were significant before adjustment are listed. A numeric suffix was appended to features with identical taxonomic classifications to ensure unique names.

Supplementary Table 4: Cox regressions of gut microbiota and MACE+

Tax	Model	Estimate	Lower CI	Higher CI	p-value	padj
Oscillospiraceae UCG-005 spp..1	Adjusted for age	0.883	0.818	0.953	0.001	0.031
Lachnospiraceae spp..2	Adjusted for age	0.870	0.804	0.941	0.001	0.031
Oscillospiraceae UCG-002 spp.	Adjusted for age	0.882	0.816	0.953	0.002	0.032
Roseburia spp.	Adjusted for age	0.899	0.830	0.974	0.009	0.077
Roseburia hominis	Adjusted for age	0.851	0.776	0.933	0.001	0.031
Lachnospiraceae spp..7	Adjusted for age	0.910	0.833	0.994	0.036	0.137
Incertae Sedis spp.	Adjusted for age	0.901	0.820	0.990	0.029	0.129
Christensenellaceae R-7 group spp.	Adjusted for age	0.877	0.807	0.954	0.002	0.036
Firmicutes spp..1	Adjusted for age	0.902	0.830	0.982	0.017	0.104
Bifidobacterium bifidum	Adjusted for age	1.081	1.008	1.161	0.030	0.129
Oscillospiraceae UCG-003 spp..1	Adjusted for age	0.913	0.834	1.000	0.049	0.173
Bilophila wadsworthia	Adjusted for age	0.910	0.834	0.992	0.033	0.134
Oscillospiraceae spp.	Adjusted for age	0.887	0.812	0.969	0.008	0.074
[Eubacterium] xylanophilum group spp.	Adjusted for age	0.807	0.740	0.881	0.000	0.000
Lachnospira spp.	Adjusted for age	0.878	0.805	0.959	0.004	0.040
Lachnospiraceae spp..1	Adjusted for age	0.847	0.769	0.934	0.001	0.031
Alistipes shahii	Adjusted for age	0.876	0.800	0.958	0.004	0.040
Oscillospiraceae UCG-005 spp..3	Adjusted for age	0.912	0.833	0.998	0.045	0.160
Terrisporobacter mayombei	Adjusted for age	0.902	0.821	0.990	0.030	0.129
Clostridium sensu stricto 1 spp.	Adjusted for age	0.893	0.808	0.987	0.027	0.129
Alistipes obesi	Adjusted for age	0.870	0.789	0.958	0.005	0.047
Odoribacter splanchnicus	Adjusted for age	0.851	0.763	0.949	0.004	0.040
Lachnospiraceae spp..4	Adjusted for age	0.886	0.806	0.974	0.012	0.090
Subdoligranulum spp.	Adjusted for age	0.911	0.833	0.995	0.038	0.145
Lachnospiraceae spp..6	Adjusted for age	0.903	0.823	0.991	0.031	0.129
Lachnospiraceae spp.	Adjusted for age	0.839	0.748	0.941	0.003	0.040
Lachnospiraceae spp..5	Adjusted for age	0.888	0.799	0.987	0.028	0.129
Clostridia UCG-014 spp.	Adjusted for age	0.884	0.800	0.978	0.016	0.104
Mogibacterium spp.	Adjusted for age	1.109	1.018	1.208	0.017	0.104
Blautia obeum	Adjusted for age	0.881	0.802	0.968	0.008	0.075
Colidextribacter spp.	Adjusted for age	0.851	0.768	0.943	0.002	0.036
Oscillospiraceae UCG-005 spp..2	Adjusted for age	0.885	0.795	0.987	0.027	0.129
Akkermansia muciniphila	Adjusted for age	0.898	0.840	0.959	0.001	0.031
Ruminococcus bicirculans	Adjusted for age	0.925	0.871	0.983	0.011	0.087
Lachnospiraceae GCA-900066575 spp.	Adjusted for age	0.815	0.723	0.918	0.001	0.031
Marvinbryantia spp.	Adjusted for age	0.866	0.772	0.971	0.014	0.101
Oscillospiraceae UCG-003 spp.	Adjusted for age	0.863	0.773	0.964	0.009	0.077

Parabacteroides distasonis	Adjusted for age	1.111	1.013	1.218	0.026	0.129
Coprococcus comes	Adjusted for age	0.910	0.835	0.991	0.031	0.129
Lachnospiraceae ND3007 group spp.	Adjusted for age	0.875	0.807	0.949	0.001	0.031
Firmicutes spp.	Adjusted for age	0.889	0.794	0.995	0.040	0.147
Oscillospiraceae UCG-005 spp.	Adjusted for age	0.831	0.734	0.941	0.003	0.040
Romboutsia ilealis/timonensis	Adjusted for age	0.913	0.849	0.982	0.015	0.103
Allisonella histaminiformans	Adjusted for age	1.127	1.015	1.250	0.025	0.129
Oscillospiraceae UCG-002 spp..1	Adjusted for age	0.900	0.843	0.960	0.001	0.031
Oscillospirales UCG-010 spp.	Adjusted for age	0.863	0.766	0.972	0.015	0.104
Faecalibacterium spp.	Adjusted for age	0.931	0.871	0.995	0.035	0.137
Christensenellaceae R-7 group spp..1	Adjusted for age	0.933	0.877	0.992	0.026	0.129
Erysipelotrichaceae UCG-003 bacterium	Adjusted for age	0.904	0.846	0.967	0.003	0.040
Coprococcus eutactus.1	Adjusted for age	0.927	0.871	0.988	0.019	0.108
Faecalibacterium prausnitzii	Adjusted for age	0.917	0.847	0.993	0.034	0.136
Coprococcus eutactus	Adjusted for age	0.921	0.861	0.986	0.017	0.104
Alistipes putredinis	Adjusted for age	0.924	0.866	0.986	0.018	0.104
Oscillospiraceae UCG-005 spp..4	Adjusted for age	0.914	0.852	0.980	0.011	0.087
[Eubacterium] eligens group spp.	Adjusted for age	0.888	0.828	0.953	0.001	0.031
Bacteroides massiliensis	Adjusted for age	0.923	0.863	0.988	0.021	0.113
Clostridium sensu stricto 1 spp..1	Adjusted for age	0.927	0.861	0.997	0.042	0.151
Clostridium sensu stricto 1 celatum/disporicum/saudiense	Adjusted for age	0.922	0.855	0.995	0.036	0.137
[Eubacterium] eligens group spp..1	Adjusted for age	0.920	0.858	0.986	0.018	0.106
Lachnospiraceae spp..3	Adjusted for age	0.880	0.810	0.957	0.003	0.040
Monoglobus spp.	Adjusted for age	0.906	0.830	0.989	0.028	0.129
[Ruminococcus] gnavus group spp.	Adjusted for age	1.103	1.033	1.178	0.004	0.040
Oscillospiraceae UCG-005 spp..1	Adjusted for age, sex, BMI, smoking and alcohol	0.907	0.839	0.980	0.014	0.282
Lachnospiraceae spp..4	Adjusted for age, sex, BMI, smoking and alcohol	0.918	0.847	0.995	0.037	0.300
Oscillospiraceae UCG-002 spp.	Adjusted for age, sex, BMI, smoking and alcohol	0.911	0.841	0.986	0.021	0.282
Roseburia spp.	Adjusted for age, sex, BMI, smoking and alcohol	0.913	0.842	0.989	0.025	0.282
Roseburia hominis	Adjusted for age, sex, BMI, smoking and alcohol	0.888	0.808	0.975	0.013	0.282
Lachnospiraceae spp..7	Adjusted for age, sex, BMI, smoking and alcohol	0.928	0.849	1.015	0.100	0.428
Incertae Sedis spp.	Adjusted for age, sex, BMI, smoking and alcohol	0.928	0.844	1.021	0.127	0.434

Christensenellaceae R-7 group spp.	Adjusted for age, sex, BMI, smoking and alcohol	0.902	0.828	0.982	0.017	0.282
Firmicutes spp..1	Adjusted for age, sex, BMI, smoking and alcohol	0.938	0.862	1.021	0.140	0.443
Bifidobacterium bifidum	Adjusted for age, sex, BMI, smoking and alcohol	1.066	0.992	1.144	0.081	0.376
Oscillospiraceae UCG-003 spp..1	Adjusted for age, sex, BMI, smoking and alcohol	0.942	0.860	1.033	0.205	0.532
Bilophila wadsworthia	Adjusted for age, sex, BMI, smoking and alcohol	0.933	0.855	1.017	0.116	0.434
Oscillospiraceae spp.	Adjusted for age, sex, BMI, smoking and alcohol	0.907	0.830	0.991	0.031	0.282
[Eubacterium] xylanophilum group spp.	Adjusted for age, sex, BMI, smoking and alcohol	0.845	0.774	0.923	0.000	0.039
Lachnospira spp.	Adjusted for age, sex, BMI, smoking and alcohol	0.920	0.842	1.004	0.062	0.341
Lachnospiraceae spp..1	Adjusted for age, sex, BMI, smoking and alcohol	0.864	0.783	0.953	0.004	0.260
Alistipes shahii	Adjusted for age, sex, BMI, smoking and alcohol	0.914	0.834	1.003	0.059	0.336
Oscillospiraceae UCG-005 spp..4	Adjusted for age, sex, BMI, smoking and alcohol	0.942	0.860	1.032	0.203	0.532
Terrisporobacter mayombei	Adjusted for age, sex, BMI, smoking and alcohol	0.912	0.830	1.002	0.055	0.324
Clostridium sensu stricto 1 spp.	Adjusted for age, sex, BMI, smoking and alcohol	0.906	0.821	0.999	0.049	0.324
Alistipes obesi	Adjusted for age, sex, BMI, smoking and alcohol	0.910	0.824	1.006	0.065	0.342
Odoribacter splanchnicus	Adjusted for age, sex, BMI, smoking and alcohol	0.893	0.800	0.997	0.044	0.309
Lachnospiraceae spp..2	Adjusted for age, sex, BMI, smoking and alcohol	0.897	0.816	0.986	0.024	0.282
Subdoligranulum spp.	Adjusted for age, sex, BMI, smoking and alcohol	0.910	0.832	0.996	0.040	0.301
Lachnospiraceae spp..5	Adjusted for age, sex, BMI, smoking and alcohol	0.918	0.836	1.008	0.072	0.350
Lachnospiraceae spp.	Adjusted for age, sex, BMI, smoking and alcohol	0.851	0.758	0.956	0.007	0.282
Lachnospiraceae spp..6	Adjusted for age, sex, BMI, smoking and alcohol	0.919	0.825	1.023	0.122	0.434
Clostridia UCG-014 spp.	Adjusted for age, sex, BMI, smoking and alcohol	0.899	0.813	0.993	0.036	0.300
Mogibacterium spp.	Adjusted for age, sex, BMI, smoking and alcohol	1.071	0.982	1.167	0.119	0.434

Blautia obeum	Adjusted for age, sex, BMI, smoking and alcohol	0.901	0.819	0.992	0.034	0.300
Colidextribacter spp.	Adjusted for age, sex, BMI, smoking and alcohol	0.885	0.797	0.983	0.022	0.282
Oscillospiraceae UCG-005 spp..2	Adjusted for age, sex, BMI, smoking and alcohol	0.911	0.816	1.016	0.094	0.408
Akkermansia muciniphila	Adjusted for age, sex, BMI, smoking and alcohol	0.928	0.867	0.993	0.031	0.282
Ruminococcus bicirculans	Adjusted for age, sex, BMI, smoking and alcohol	0.954	0.899	1.014	0.130	0.434
Lachnospiraceae GCA-900066575 spp.	Adjusted for age, sex, BMI, smoking and alcohol	0.833	0.738	0.940	0.003	0.260
Marvinbryantia spp.	Adjusted for age, sex, BMI, smoking and alcohol	0.900	0.800	1.013	0.080	0.376
Oscillospiraceae UCG-003 spp.	Adjusted for age, sex, BMI, smoking and alcohol	0.880	0.787	0.983	0.024	0.282
Parabacteroides distasonis	Adjusted for age, sex, BMI, smoking and alcohol	1.137	1.036	1.247	0.007	0.282
Coprococcus comes	Adjusted for age, sex, BMI, smoking and alcohol	0.904	0.830	0.984	0.020	0.282
Lachnospiraceae ND3007 group spp.	Adjusted for age, sex, BMI, smoking and alcohol	0.897	0.825	0.976	0.011	0.282
Firmicutes spp.	Adjusted for age, sex, BMI, smoking and alcohol	0.900	0.803	1.009	0.071	0.350
Oscillospiraceae UCG-005 spp.	Adjusted for age, sex, BMI, smoking and alcohol	0.865	0.763	0.980	0.023	0.282
Romboutsia ilealis/timonensis	Adjusted for age, sex, BMI, smoking and alcohol	0.931	0.865	1.001	0.054	0.324
Allisonella histaminiformans	Adjusted for age, sex, BMI, smoking and alcohol	1.065	0.958	1.185	0.243	0.563
Oscillospiraceae UCG-002 spp..1	Adjusted for age, sex, BMI, smoking and alcohol	0.928	0.869	0.992	0.028	0.282
Oscillospirales UCG-010 spp.	Adjusted for age, sex, BMI, smoking and alcohol	0.909	0.805	1.026	0.123	0.434
Faecalibacterium spp.	Adjusted for age, sex, BMI, smoking and alcohol	0.946	0.885	1.012	0.107	0.432
Christensenellaceae R-7 group spp..1	Adjusted for age, sex, BMI, smoking and alcohol	0.968	0.909	1.031	0.318	0.623
Erysipelotrichaceae UCG-003 bacterium	Adjusted for age, sex, BMI, smoking and alcohol	0.922	0.862	0.986	0.018	0.282
Coprococcus eutactus	Adjusted for age, sex, BMI, smoking and alcohol	0.936	0.879	0.997	0.041	0.301
Faecalibacterium prausnitzii	Adjusted for age, sex, BMI, smoking and alcohol	0.923	0.852	1.000	0.049	0.324

Coprococcus eutactus.1	Adjusted for age, sex, BMI, smoking and alcohol	0.939	0.878	1.005	0.070	0.350
Alistipes putredinis	Adjusted for age, sex, BMI, smoking and alcohol	0.950	0.888	1.016	0.133	0.434
Oscillospiraceae UCG-005 spp..3	Adjusted for age, sex, BMI, smoking and alcohol	0.940	0.875	1.009	0.088	0.391
[Eubacterium] eligens group spp.	Adjusted for age, sex, BMI, smoking and alcohol	0.914	0.852	0.982	0.014	0.282
Bacteroides massiliensis	Adjusted for age, sex, BMI, smoking and alcohol	0.928	0.867	0.993	0.030	0.282
Clostridium sensu stricto 1 spp..1	Adjusted for age, sex, BMI, smoking and alcohol	0.945	0.878	1.017	0.132	0.434
Clostridium sensu stricto 1 celatum/disporicum/saudiense	Adjusted for age, sex, BMI, smoking and alcohol	0.924	0.857	0.997	0.041	0.301
[Eubacterium] eligens group spp..1	Adjusted for age, sex, BMI, smoking and alcohol	0.950	0.884	1.020	0.157	0.476
Lachnospiraceae spp..3	Adjusted for age, sex, BMI, smoking and alcohol	0.910	0.836	0.991	0.030	0.282
Monoglobus spp.	Adjusted for age, sex, BMI, smoking and alcohol	0.951	0.870	1.038	0.261	0.575
[Ruminococcus] gnavus group spp.	Adjusted for age, sex, BMI, smoking and alcohol	1.088	1.019	1.163	0.012	0.282

Coefficients resulting from Cox regression models adjusted for age, including the hazard ratios (HR) and 95%-confidence intervals (CI). P-values were adjusted using the false discovery ratio. All amplicon sequence variants (ASVs) that were significant before adjustment are listed. A numeric suffix was appended to features with identical taxonomic classifications to ensure unique names.

Supplementary Table 5: Cox regressions of dietary data and MACE

Food group	Outcome	Estimate	Lower CI	Higher CI	p-value
Fiber	MACE	1.00	0.96	1.03	0.83
Protein	MACE	1.00	0.99	1.01	0.67
Fat	MACE	1.00	1.00	1.01	0.42
Carb	MACE	1.00	1.00	1.00	0.92
Sodium	MACE	4.96	0.62	39.78	0.13
Animal protein	MACE	1.00	0.99	1.01	0.91
Total calories	MACE	1.00	1.00	1.00	0.58
Fiber	MACE+	0.99	0.96	1.02	0.64
Protein	MACE+	0.99	0.99	1.00	0.84
Fat	MACE+	1.00	0.99	1.00	0.79
Carb	MACE+	0.99	0.99	1.00	0.61
Sodium	MACE+	1.69	0.33	8.47	0.52
Animal protein	MACE+	1.00	0.98	1.00	0.70
Total calories	MACE+	1.00	0.99	1.00	0.88

Cox regression models predicting MACE and MACE+ from dietary data. Sodium intake was log10-transformed. None of the models were significant. Dietary data was available for N=1292, except sodium intake, which was available for N=1027.

Supplementary Table 6: Logistic regressions of gut microbiota and new-onset diabetes

Tax	Model	OR	Lower CI	Upper CI	p-value	padj
Lachnospiraceae spp.	Adjusted for age	0.845	0.779	0.916	0.000	0.002
Lachnospiraceae spp.	Adjusted for age, sex, BMI, smoking, alcohol	0.872	0.800	0.948	0.001	0.019
Lachnospiraceae spp..1	Adjusted for age	0.891	0.819	0.969	0.007	0.014
Lachnospiraceae spp..1	Adjusted for age, sex, BMI, smoking, alcohol	0.888	0.815	0.968	0.007	0.032
Lachnospiraceae spp..2	Adjusted for age	0.857	0.767	0.951	0.005	0.011
Lachnospiraceae spp..2	Adjusted for age, sex, BMI, smoking, alcohol	0.886	0.791	0.986	0.030	0.063
Lachnospiraceae UCG-004 spp.	Adjusted for age	1.287	1.127	1.478	0.000	0.003
Lachnospiraceae UCG-004 spp.	Adjusted for age, sex, BMI, smoking, alcohol	1.289	1.123	1.489	0.000	0.016
Lachnoclostridium spp.	Adjusted for age	1.163	1.018	1.334	0.029	0.036
Lachnoclostridium spp.	Adjusted for age, sex, BMI, smoking, alcohol	1.114	0.971	1.284	0.130	0.153
Marvinbryantia spp.	Adjusted for age	0.807	0.715	0.909	0.000	0.003
Marvinbryantia spp.	Adjusted for age, sex, BMI, smoking, alcohol	0.824	0.726	0.932	0.002	0.019
Coprococcus catus	Adjusted for age	0.893	0.811	0.991	0.027	0.036
Coprococcus catus	Adjusted for age, sex, BMI, smoking, alcohol	0.928	0.835	1.040	0.180	0.193
Lachnospiraceae spp..3	Adjusted for age	0.826	0.720	0.949	0.007	0.014
Lachnospiraceae spp..3	Adjusted for age, sex, BMI, smoking, alcohol	0.863	0.747	0.997	0.044	0.076
Lachnospiraceae GCA-900066575 spp.	Adjusted for age	0.864	0.763	0.980	0.023	0.033
Lachnospiraceae GCA-900066575 spp.	Adjusted for age, sex, BMI, smoking, alcohol	0.878	0.771	1.002	0.052	0.085
[Eubacterium] ruminantium group spp.	Adjusted for age	0.927	0.866	0.991	0.028	0.036
[Eubacterium] ruminantium group spp.	Adjusted for age, sex, BMI, smoking, alcohol	0.926	0.863	0.992	0.031	0.063
Lachnospiraceae ND3007 group spp.	Adjusted for age	0.836	0.767	0.916	0.000	0.002
Lachnospiraceae ND3007 group spp.	Adjusted for age, sex, BMI, smoking, alcohol	0.845	0.771	0.931	0.000	0.016
[Eubacterium] xylanophilum group spp.	Adjusted for age	0.870	0.798	0.948	0.002	0.006
[Eubacterium] xylanophilum group spp.	Adjusted for age, sex, BMI, smoking, alcohol	0.906	0.828	0.991	0.031	0.063
Oscillospiraceae NK4A214 group spp.	Adjusted for age	0.846	0.767	0.927	0.001	0.003
Oscillospiraceae NK4A214 group spp.	Adjusted for age, sex, BMI, smoking, alcohol	0.882	0.797	0.969	0.011	0.037
Oscillospiraceae NK4A214 group spp..1	Adjusted for age	0.861	0.768	0.957	0.007	0.015
Oscillospiraceae NK4A214 group spp..1	Adjusted for age, sex, BMI, smoking, alcohol	0.886	0.789	0.987	0.034	0.068
Oscillospiraceae UCG-005 spp.	Adjusted for age	0.881	0.820	0.947	0.001	0.003
Oscillospiraceae UCG-005 spp.	Adjusted for age, sex, BMI, smoking, alcohol	0.907	0.842	0.979	0.012	0.037
Oscillospiraceae spp.	Adjusted for age	0.863	0.787	0.945	0.002	0.006
Oscillospiraceae spp.	Adjusted for age, sex, BMI, smoking, alcohol	0.875	0.797	0.962	0.006	0.032

Oscillospiraceae UCG-005 spp..1	Adjusted for age	0.909	0.842	0.980	0.014	0.023
Oscillospiraceae UCG-005 spp..1	Adjusted for age, sex, BMI, smoking, alcohol	0.928	0.856	1.005	0.069	0.099
Oscillospiraceae UCG-005 spp..2	Adjusted for age	0.779	0.684	0.883	0.000	0.002
Oscillospiraceae UCG-005 spp..2	Adjusted for age, sex, BMI, smoking, alcohol	0.828	0.724	0.944	0.005	0.032
Oscillospiraceae UCG-002 spp.	Adjusted for age	0.847	0.767	0.928	0.001	0.003
Oscillospiraceae UCG-002 spp.	Adjusted for age, sex, BMI, smoking, alcohol	0.886	0.800	0.974	0.015	0.041
Oscillospiraceae UCG-002 spp..1	Adjusted for age	0.888	0.831	0.950	0.001	0.003
Oscillospiraceae UCG-002 spp..1	Adjusted for age, sex, BMI, smoking, alcohol	0.904	0.842	0.970	0.005	0.032
Oscillospiraceae UCG-002 spp..2	Adjusted for age	0.927	0.857	0.998	0.050	0.050
Oscillospiraceae UCG-002 spp..2	Adjusted for age, sex, BMI, smoking, alcohol	0.949	0.876	1.024	0.182	0.193
Oscillospiraceae UCG-002 spp..3	Adjusted for age	0.913	0.848	0.986	0.018	0.028
Oscillospiraceae UCG-002 spp..3	Adjusted for age, sex, BMI, smoking, alcohol	0.907	0.840	0.982	0.014	0.041
Flavonifractor plautii	Adjusted for age	1.171	1.058	1.293	0.002	0.007
Flavonifractor plautii	Adjusted for age, sex, BMI, smoking, alcohol	1.176	1.058	1.306	0.003	0.019
Intestinimonas spp.	Adjusted for age	0.846	0.751	0.951	0.005	0.012
Intestinimonas spp.	Adjusted for age, sex, BMI, smoking, alcohol	0.880	0.778	0.996	0.043	0.076
Colidextribacter massiliensis	Adjusted for age	0.805	0.708	0.910	0.001	0.003
Colidextribacter massiliensis	Adjusted for age, sex, BMI, smoking, alcohol	0.841	0.737	0.955	0.009	0.034
Colidextribacter spp.	Adjusted for age	0.861	0.777	0.951	0.004	0.010
Colidextribacter spp.	Adjusted for age, sex, BMI, smoking, alcohol	0.899	0.809	0.996	0.044	0.076
Oscillospirales UCG-010 spp.	Adjusted for age	0.882	0.783	0.987	0.033	0.038
Oscillospirales UCG-010 spp.	Adjusted for age, sex, BMI, smoking, alcohol	0.913	0.808	1.025	0.131	0.153
[Eubacterium] siraeum group spp.	Adjusted for age	0.902	0.811	0.997	0.050	0.050
[Eubacterium] siraeum group spp.	Adjusted for age, sex, BMI, smoking, alcohol	0.941	0.845	1.042	0.258	0.258
[Eubacterium] siraeum group spp..1	Adjusted for age	0.897	0.832	0.965	0.004	0.011
[Eubacterium] siraeum group spp..1	Adjusted for age, sex, BMI, smoking, alcohol	0.912	0.843	0.984	0.019	0.047
Ruminococcaceae spp.	Adjusted for age	0.878	0.805	0.955	0.003	0.008
Ruminococcaceae spp.	Adjusted for age, sex, BMI, smoking, alcohol	0.898	0.822	0.979	0.016	0.043
Subdoligranulum spp.	Adjusted for age	0.910	0.846	0.982	0.013	0.023
Subdoligranulum spp.	Adjusted for age, sex, BMI, smoking, alcohol	0.921	0.852	0.998	0.039	0.073
Butyricicoccus spp.	Adjusted for age	0.913	0.844	0.987	0.022	0.033
Butyricicoccus spp.	Adjusted for age, sex, BMI, smoking, alcohol	0.917	0.846	0.994	0.036	0.069
Ruminococcaceae spp..1	Adjusted for age	0.842	0.764	0.929	0.001	0.003
Ruminococcaceae spp..1	Adjusted for age, sex, BMI, smoking, alcohol	0.873	0.789	0.968	0.009	0.034
[Eubacterium] coprostanoligenes group spp.	Adjusted for age	0.865	0.768	0.968	0.014	0.023

[Eubacterium] coprostanoligenes group spp.	Adjusted for age, sex, BMI, smoking, alcohol	0.895	0.793	1.005	0.066	0.099
Mogibacterium spp.	Adjusted for age	1.139	1.042	1.242	0.004	0.010
Mogibacterium spp.	Adjusted for age, sex, BMI, smoking, alcohol	1.097	1.000	1.201	0.046	0.077
Romboutsia ilealis/timonensis	Adjusted for age	0.900	0.831	0.976	0.010	0.019
Romboutsia ilealis/timonensis	Adjusted for age, sex, BMI, smoking, alcohol	0.911	0.839	0.991	0.029	0.063
Alistipes putredinis	Adjusted for age	0.912	0.853	0.974	0.006	0.014
Alistipes putredinis	Adjusted for age, sex, BMI, smoking, alcohol	0.935	0.872	1.001	0.055	0.087
Alistipes shahii	Adjusted for age	0.901	0.823	0.987	0.025	0.033
Alistipes shahii	Adjusted for age, sex, BMI, smoking, alcohol	0.931	0.846	1.024	0.140	0.159
Alistipes obesi	Adjusted for age	0.906	0.822	0.998	0.047	0.049
Alistipes obesi	Adjusted for age, sex, BMI, smoking, alcohol	0.932	0.841	1.033	0.181	0.193
Parabacteroides distasonis	Adjusted for age	0.888	0.798	0.983	0.025	0.033
Parabacteroides distasonis	Adjusted for age, sex, BMI, smoking, alcohol	0.906	0.812	1.006	0.073	0.102
Bacteroides uniformis	Adjusted for age	0.922	0.860	0.989	0.023	0.033
Bacteroides uniformis	Adjusted for age, sex, BMI, smoking, alcohol	0.939	0.873	1.009	0.088	0.115
Bacteroides dorei/fragilis	Adjusted for age	1.075	1.003	1.150	0.037	0.040
Bacteroides dorei/fragilis	Adjusted for age, sex, BMI, smoking, alcohol	1.097	1.020	1.176	0.011	0.037
Bacteroides fragilis/ovatus	Adjusted for age	1.098	1.015	1.188	0.020	0.031
Bacteroides fragilis/ovatus	Adjusted for age, sex, BMI, smoking, alcohol	1.112	1.023	1.208	0.013	0.038
Odoribacter splanchnicus	Adjusted for age	0.882	0.788	0.990	0.031	0.038
Odoribacter splanchnicus	Adjusted for age, sex, BMI, smoking, alcohol	0.918	0.816	1.035	0.159	0.177
Akkermansia muciniphila	Adjusted for age	0.889	0.830	0.950	0.001	0.003
Akkermansia muciniphila	Adjusted for age, sex, BMI, smoking, alcohol	0.912	0.849	0.976	0.009	0.034
Olsenella spp.	Adjusted for age	1.110	1.002	1.227	0.044	0.046
Olsenella spp.	Adjusted for age, sex, BMI, smoking, alcohol	1.073	0.964	1.191	0.194	0.203
Holdemanella biformis	Adjusted for age	1.069	1.004	1.137	0.033	0.038
Holdemanella biformis	Adjusted for age, sex, BMI, smoking, alcohol	1.058	0.992	1.127	0.085	0.114
Erysipelotrichaceae UCG-003 bacterium	Adjusted for age	0.877	0.817	0.941	0.000	0.003
Erysipelotrichaceae UCG-003 bacterium	Adjusted for age, sex, BMI, smoking, alcohol	0.906	0.842	0.976	0.009	0.034
Phascolarctobacterium faecium	Adjusted for age	0.914	0.839	0.990	0.033	0.038
Phascolarctobacterium faecium	Adjusted for age, sex, BMI, smoking, alcohol	0.935	0.857	1.014	0.116	0.141
Dialister spp.	Adjusted for age	1.071	1.013	1.130	0.013	0.023
Dialister spp.	Adjusted for age, sex, BMI, smoking, alcohol	1.049	0.991	1.109	0.094	0.120
Clostridia spp.	Adjusted for age	0.903	0.834	0.974	0.010	0.019
Clostridia spp.	Adjusted for age, sex, BMI, smoking, alcohol	0.935	0.862	1.010	0.096	0.121
Clostridia spp..1	Adjusted for age	0.915	0.841	0.991	0.034	0.038

Clostridia spp..1	Adjusted for age, sex, BMI, smoking, alcohol	0.953	0.874	1.034	0.258	0.258
Christensenellaceae R-7 group spp.	Adjusted for age	0.876	0.772	0.988	0.034	0.038
Christensenellaceae R-7 group spp.	Adjusted for age, sex, BMI, smoking, alcohol	0.908	0.797	1.028	0.136	0.156
Christensenellaceae R-7 group spp..1	Adjusted for age	0.907	0.831	0.992	0.031	0.038
Christensenellaceae R-7 group spp..1	Adjusted for age, sex, BMI, smoking, alcohol	0.917	0.836	1.008	0.069	0.099
Christensenellaceae R-7 group spp..2	Adjusted for age	0.877	0.806	0.952	0.002	0.006
Christensenellaceae R-7 group spp..2	Adjusted for age, sex, BMI, smoking, alcohol	0.889	0.815	0.967	0.007	0.032
Christensenellaceae R-7 group spp..3	Adjusted for age	0.889	0.790	0.993	0.042	0.045
Christensenellaceae R-7 group spp..3	Adjusted for age, sex, BMI, smoking, alcohol	0.907	0.804	1.016	0.100	0.124
Clostridia UCG-014 spp.	Adjusted for age	0.913	0.832	0.998	0.050	0.050
Clostridia UCG-014 spp.	Adjusted for age, sex, BMI, smoking, alcohol	0.941	0.855	1.031	0.197	0.203
Monoglobus spp.	Adjusted for age	0.879	0.801	0.966	0.006	0.014
Monoglobus spp.	Adjusted for age, sex, BMI, smoking, alcohol	0.918	0.835	1.012	0.082	0.114
[Eubacterium] hallii group spp.	Adjusted for age	0.882	0.793	0.987	0.024	0.033
[Eubacterium] hallii group spp.	Adjusted for age, sex, BMI, smoking, alcohol	0.894	0.799	1.006	0.055	0.087
Lachnospiraceae spp..4	Adjusted for age	0.810	0.726	0.901	0.000	0.002
Lachnospiraceae spp..4	Adjusted for age, sex, BMI, smoking, alcohol	0.838	0.749	0.936	0.002	0.019
[Ruminococcus] gnavus group spp.	Adjusted for age	1.115	1.037	1.195	0.003	0.008
[Ruminococcus] gnavus group spp.	Adjusted for age, sex, BMI, smoking, alcohol	1.133	1.051	1.219	0.001	0.016
Marvinbryantia spp..1	Adjusted for age	0.840	0.745	0.942	0.003	0.010
Marvinbryantia spp..1	Adjusted for age, sex, BMI, smoking, alcohol	0.872	0.770	0.984	0.029	0.063
Marvinbryantia spp..2	Adjusted for age	0.841	0.739	0.959	0.009	0.017
Marvinbryantia spp..2	Adjusted for age, sex, BMI, smoking, alcohol	0.852	0.744	0.978	0.021	0.052
Blautia obeum	Adjusted for age	0.858	0.774	0.955	0.004	0.011
Blautia obeum	Adjusted for age, sex, BMI, smoking, alcohol	0.881	0.789	0.989	0.028	0.063
Blautia spp.	Adjusted for age	0.838	0.759	0.928	0.001	0.003
Blautia spp.	Adjusted for age, sex, BMI, smoking, alcohol	0.835	0.752	0.931	0.001	0.016
Blautia spp..1	Adjusted for age	0.799	0.704	0.905	0.000	0.003
Blautia spp..1	Adjusted for age, sex, BMI, smoking, alcohol	0.812	0.712	0.925	0.002	0.019
Blautia faecis	Adjusted for age	0.858	0.757	0.981	0.021	0.032
Blautia faecis	Adjusted for age, sex, BMI, smoking, alcohol	0.885	0.772	1.022	0.086	0.114
Lachnospiraceae spp..5	Adjusted for age	0.869	0.803	0.937	0.000	0.003
Lachnospiraceae spp..5	Adjusted for age, sex, BMI, smoking, alcohol	0.897	0.828	0.969	0.006	0.032
Roseburia hominis	Adjusted for age	0.894	0.811	0.985	0.023	0.033
Roseburia hominis	Adjusted for age, sex, BMI, smoking, alcohol	0.910	0.824	1.005	0.063	0.097

Table shows coefficients resulting from logistic regression models for incident diabetes including the odds ratios (OR) and 95%-confidence intervals (CI). P-values were adjusted using the false discovery ratio. All amplicon sequence variants (ASVs) that were significant after adjustment are listed. A numeric suffix was appended to features with identical taxonomic classifications to ensure unique names.

Supplementary Table 7: Logistic regressions of gut microbiota and new-onset dyslipidemia

Tax	Model	OR	Lower CI	Upper CI	p-value	padj
Lachnospiraceae spp.	Adjusted for age	0.8918	0.8410	0.9452	0.0001	0.0027
Lachnospiraceae spp.	Adjusted for age. sex. BMI. smoking. alcohol	0.9099	0.8566	0.9661	0.0021	0.0258
Lachnospiraceae spp..1	Adjusted for age	0.8630	0.7856	0.9480	0.0021	0.0181
Lachnospiraceae spp..1	Adjusted for age. sex. BMI. smoking. alcohol	0.8720	0.7924	0.9594	0.0050	0.0381
Coprococcus catus	Adjusted for age	0.8960	0.8260	0.9748	0.0092	0.0473
Coprococcus catus	Adjusted for age. sex. BMI. smoking. alcohol	0.8968	0.8253	0.9775	0.0115	0.0730
Lachnospira spp.	Adjusted for age	0.8621	0.8073	0.9192	0.0000	0.0007
Lachnospira spp.	Adjusted for age. sex. BMI. smoking. alcohol	0.8762	0.8199	0.9351	0.0001	0.0037
Lachnospiraceae ND3007 group spp.	Adjusted for age	0.8575	0.7971	0.9240	0.0000	0.0014
Lachnospiraceae ND3007 group spp.	Adjusted for age. sex. BMI. smoking. alcohol	0.8698	0.8072	0.9389	0.0003	0.0067
Oscillospiraceae UCG-005 spp.	Adjusted for age	0.8861	0.8089	0.9698	0.0090	0.0473
Oscillospiraceae UCG-005 spp.	Adjusted for age. sex. BMI. smoking. alcohol	0.9140	0.8322	1.0032	0.0591	0.1875
Oscillospiraceae UCG-002 spp.	Adjusted for age	0.9194	0.8627	0.9780	0.0085	0.0473
Oscillospiraceae UCG-002 spp.	Adjusted for age. sex. BMI. smoking. alcohol	0.9347	0.8757	0.9960	0.0396	0.1584
Flavonifractor plautii	Adjusted for age	1.1318	1.0465	1.2232	0.0018	0.0181
Flavonifractor plautii	Adjusted for age. sex. BMI. smoking. alcohol	1.1429	1.0547	1.2377	0.0011	0.0195
Oscillospirales UCG-010 spp.	Adjusted for age	0.8844	0.8133	0.9597	0.0036	0.0255
Oscillospirales UCG-010 spp.	Adjusted for age. sex. BMI. smoking. alcohol	0.9011	0.8274	0.9793	0.0153	0.0830
Terrisporobacter mayombei	Adjusted for age	0.8581	0.7990	0.9196	0.0000	0.0009
Terrisporobacter mayombei	Adjusted for age. sex. BMI. smoking. alcohol	0.8629	0.8027	0.9257	0.0000	0.0037
Erysipelotrichaceae UCG-003 bacterium	Adjusted for age	0.9242	0.8748	0.9767	0.0050	0.0327
Erysipelotrichaceae UCG-003 bacterium	Adjusted for age. sex. BMI. smoking. alcohol	0.9345	0.8836	0.9889	0.0184	0.0938
Clostridium sensu stricto 1 spp.	Adjusted for age	0.9131	0.8639	0.9647	0.0012	0.0154
Clostridium sensu stricto 1 spp.	Adjusted for age. sex. BMI. smoking. alcohol	0.9151	0.8650	0.9675	0.0019	0.0258
Clostridium sensu stricto 1 celatum/disporicum/saudiense	Adjusted for age	0.9119	0.8592	0.9670	0.0022	0.0181
Clostridium sensu stricto 1 celatum/disporicum/saudiense	Adjusted for age. sex. BMI. smoking. alcohol	0.9124	0.8591	0.9683	0.0027	0.0273
Clostridia spp.	Adjusted for age	0.8978	0.8284	0.9707	0.0076	0.0467
Clostridia spp.	Adjusted for age. sex. BMI. smoking. alcohol	0.9015	0.8304	0.9762	0.0119	0.0730
Lachnospiraceae spp..2	Adjusted for age	0.8683	0.8023	0.9391	0.0004	0.0067
Lachnospiraceae spp..2	Adjusted for age. sex. BMI. smoking. alcohol	0.8818	0.8133	0.9557	0.0022	0.0258
[Ruminococcus] gnavus group spp.	Adjusted for age	1.1099	1.0467	1.1753	0.0004	0.0067
[Ruminococcus] gnavus group spp.	Adjusted for age. sex. BMI. smoking. alcohol	1.1214	1.0564	1.1890	0.0001	0.0044
Marvinbryantia spp.	Adjusted for age	0.8788	0.8080	0.9546	0.0024	0.0181

Marvinbryantia spp.	Adjusted for age. sex. BMI. smoking. alcohol	0.8840	0.8107	0.9627	0.0049	0.0381
Blautia obeum	Adjusted for age	0.8716	0.8020	0.9487	0.0013	0.0154
Blautia obeum	Adjusted for age. sex. BMI. smoking. alcohol	0.8796	0.8077	0.9594	0.0035	0.0318

Table shows coefficients resulting from logistic regression models for incident dyslipidemia including the odds ratios (OR) and 95%-confidence intervals (CI). P-values were adjusted using the false discovery ratio. All amplicon sequence variants (ASVs) that were significant after adjustment are listed. A numeric suffix was appended to features with identical taxonomic classifications to ensure unique names.

Supplementary Table 8: Logistic regressions of gut microbiota and new-onset hypertension

Tax	Model	OR	Lower CI	Upper CI	pvalue	padj
Lachnospiraceae UCG-001 spp.	Adjusted for age	0.917	0.859	0.977	0.008	0.015
Lachnospiraceae UCG-001 spp.	Adjusted for age. sex. BMI. smoking. alcohol	0.918	0.859	0.981	0.012	0.023
Lachnospiraceae spp.	Adjusted for age	0.847	0.798	0.899	0.000	0.000
Lachnospiraceae spp.	Adjusted for age. sex. BMI. smoking. alcohol	0.870	0.817	0.926	0.000	0.001
Lachnospiraceae spp..1	Adjusted for age	0.891	0.832	0.953	0.001	0.003
Lachnospiraceae spp..1	Adjusted for age. sex. BMI. smoking. alcohol	0.902	0.841	0.968	0.004	0.015
Lachnospiraceae spp..2	Adjusted for age	0.928	0.869	0.990	0.024	0.031
Lachnospiraceae spp..2	Adjusted for age. sex. BMI. smoking. alcohol	0.934	0.874	0.999	0.045	0.062
Lachnospiraceae UCG-004 spp.	Adjusted for age	1.110	1.013	1.216	0.024	0.031
Lachnospiraceae UCG-004 spp.	Adjusted for age. sex. BMI. smoking. alcohol	1.104	1.005	1.213	0.038	0.054
Lachnospiraceae spp..3	Adjusted for age	0.881	0.800	0.972	0.011	0.017
Lachnospiraceae spp..3	Adjusted for age. sex. BMI. smoking. alcohol	0.899	0.813	0.995	0.038	0.054
Lachnospiraceae spp..4	Adjusted for age	0.885	0.807	0.971	0.010	0.016
Lachnospiraceae spp..4	Adjusted for age. sex. BMI. smoking. alcohol	0.883	0.802	0.972	0.011	0.023
Lachnospiraceae spp..5	Adjusted for age	0.883	0.803	0.970	0.010	0.016
Lachnospiraceae spp..5	Adjusted for age. sex. BMI. smoking. alcohol	0.880	0.797	0.971	0.011	0.023
Marvinbryantia spp.	Adjusted for age	0.858	0.783	0.940	0.001	0.003
Marvinbryantia spp.	Adjusted for age. sex. BMI. smoking. alcohol	0.880	0.800	0.966	0.008	0.020
[Eubacterium] eligens group spp.	Adjusted for age	0.900	0.850	0.953	0.000	0.002
[Eubacterium] eligens group spp.	Adjusted for age. sex. BMI. smoking. alcohol	0.912	0.859	0.968	0.003	0.012
[Eubacterium] eligens group spp..1	Adjusted for age	0.920	0.869	0.975	0.004	0.010
[Eubacterium] eligens group spp..1	Adjusted for age. sex. BMI. smoking. alcohol	0.918	0.865	0.975	0.005	0.016
Coprococcus catus	Adjusted for age	0.907	0.837	0.986	0.019	0.027
Coprococcus catus	Adjusted for age. sex. BMI. smoking. alcohol	0.924	0.849	1.009	0.072	0.084
Lachnospiraceae GCA-900066575 spp.	Adjusted for age	0.870	0.790	0.959	0.005	0.010
Lachnospiraceae GCA-900066575 spp.	Adjusted for age. sex. BMI. smoking. alcohol	0.879	0.796	0.972	0.011	0.023
Lachnospira spp.	Adjusted for age	0.927	0.868	0.988	0.021	0.029
Lachnospira spp.	Adjusted for age. sex. BMI. smoking. alcohol	0.939	0.878	1.004	0.065	0.079
[Eubacterium] ruminantium group spp.	Adjusted for age	0.927	0.881	0.974	0.003	0.007
[Eubacterium] ruminantium group spp.	Adjusted for age. sex. BMI. smoking. alcohol	0.925	0.878	0.974	0.003	0.013
Lachnospiraceae ND3007 group spp.	Adjusted for age	0.906	0.840	0.980	0.012	0.019
Lachnospiraceae ND3007 group spp.	Adjusted for age. sex. BMI. smoking. alcohol	0.915	0.845	0.992	0.029	0.043
[Eubacterium] xylanophilum group spp.	Adjusted for age	0.894	0.837	0.954	0.001	0.003

[Eubacterium] xylanophilum group spp.	Adjusted for age. sex. BMI. smoking. alcohol	0.918	0.858	0.983	0.014	0.027
Oscillospiraceae NK4A214 group spp.	Adjusted for age	0.874	0.819	0.931	0.000	0.001
Oscillospiraceae NK4A214 group spp.	Adjusted for age. sex. BMI. smoking. alcohol	0.899	0.841	0.959	0.001	0.010
Oscillospiraceae NK4A214 group spp..1	Adjusted for age	0.862	0.797	0.930	0.000	0.001
Oscillospiraceae NK4A214 group spp..1	Adjusted for age. sex. BMI. smoking. alcohol	0.871	0.803	0.941	0.001	0.010
Oscillospiraceae NK4A214 group spp..2	Adjusted for age	0.916	0.867	0.968	0.002	0.005
Oscillospiraceae NK4A214 group spp..2	Adjusted for age. sex. BMI. smoking. alcohol	0.914	0.864	0.967	0.002	0.011
Oscillospiraceae UCG-005 spp.	Adjusted for age	0.902	0.853	0.954	0.000	0.002
Oscillospiraceae UCG-005 spp.	Adjusted for age. sex. BMI. smoking. alcohol	0.920	0.869	0.975	0.005	0.016
Oscillospiraceae spp.	Adjusted for age	0.901	0.839	0.966	0.004	0.008
Oscillospiraceae spp.	Adjusted for age. sex. BMI. smoking. alcohol	0.915	0.851	0.983	0.016	0.027
Intestinimonas spp.	Adjusted for age	0.940	0.884	0.998	0.045	0.046
Intestinimonas spp.	Adjusted for age. sex. BMI. smoking. alcohol	0.926	0.869	0.985	0.016	0.027
Oscillospiraceae UCG-005 spp..1	Adjusted for age	0.890	0.840	0.943	0.000	0.001
Oscillospiraceae UCG-005 spp..1	Adjusted for age. sex. BMI. smoking. alcohol	0.905	0.852	0.960	0.001	0.010
Oscillospiraceae UCG-005 spp..2	Adjusted for age	0.807	0.735	0.886	0.000	0.000
Oscillospiraceae UCG-005 spp..2	Adjusted for age. sex. BMI. smoking. alcohol	0.853	0.774	0.940	0.001	0.010
Oscillospiraceae UCG-005 spp..3	Adjusted for age	0.921	0.851	0.996	0.041	0.043
Oscillospiraceae UCG-005 spp..3	Adjusted for age. sex. BMI. smoking. alcohol	0.942	0.868	1.020	0.144	0.153
Oscillospiraceae spp..1	Adjusted for age	0.904	0.840	0.973	0.007	0.014
Oscillospiraceae spp..1	Adjusted for age. sex. BMI. smoking. alcohol	0.915	0.848	0.986	0.021	0.034
Oscillospiraceae UCG-002 spp.	Adjusted for age	0.924	0.863	0.989	0.024	0.031
Oscillospiraceae UCG-002 spp.	Adjusted for age. sex. BMI. smoking. alcohol	0.924	0.861	0.991	0.027	0.042
Oscillospiraceae UCG-002 spp..1	Adjusted for age	0.913	0.854	0.974	0.006	0.012
Oscillospiraceae UCG-002 spp..1	Adjusted for age. sex. BMI. smoking. alcohol	0.937	0.875	1.001	0.057	0.071
Oscillospiraceae UCG-002 spp..2	Adjusted for age	0.907	0.860	0.956	0.000	0.002
Oscillospiraceae UCG-002 spp..2	Adjusted for age. sex. BMI. smoking. alcohol	0.920	0.871	0.972	0.003	0.012
Oscillospiraceae UCG-002 spp..3	Adjusted for age	0.944	0.892	0.999	0.047	0.047
Oscillospiraceae UCG-002 spp..3	Adjusted for age. sex. BMI. smoking. alcohol	0.968	0.913	1.025	0.270	0.273
Oscillospiraceae UCG-002 spp..4	Adjusted for age	0.895	0.841	0.952	0.000	0.002
Oscillospiraceae UCG-002 spp..4	Adjusted for age. sex. BMI. smoking. alcohol	0.912	0.855	0.972	0.005	0.016
Flavonifractor plautii	Adjusted for age	1.150	1.064	1.244	0.000	0.002
Flavonifractor plautii	Adjusted for age. sex. BMI. smoking. alcohol	1.167	1.076	1.266	0.000	0.004
Intestinimonas spp..1	Adjusted for age	0.905	0.828	0.990	0.029	0.034
Intestinimonas spp..1	Adjusted for age. sex. BMI. smoking. alcohol	0.948	0.863	1.042	0.269	0.273
Colidextribacter massiliensis	Adjusted for age	0.894	0.818	0.976	0.013	0.020
Colidextribacter massiliensis	Adjusted for age. sex. BMI. smoking. alcohol	0.917	0.837	1.005	0.064	0.079

Colidextribacter spp.	Adjusted for age	0.859	0.795	0.926	0.000	0.001
Colidextribacter spp.	Adjusted for age. sex. BMI. smoking. alcohol	0.881	0.814	0.952	0.001	0.010
Oscillospirales UCG-010 spp.	Adjusted for age	0.879	0.806	0.956	0.003	0.007
Oscillospirales UCG-010 spp.	Adjusted for age. sex. BMI. smoking. alcohol	0.898	0.822	0.979	0.015	0.027
[Eubacterium] siraeum group spp.	Adjusted for age	0.864	0.798	0.932	0.000	0.002
[Eubacterium] siraeum group spp.	Adjusted for age. sex. BMI. smoking. alcohol	0.888	0.819	0.960	0.003	0.013
[Eubacterium] siraeum group spp..1	Adjusted for age	0.943	0.892	0.996	0.038	0.040
[Eubacterium] siraeum group spp..1	Adjusted for age. sex. BMI. smoking. alcohol	0.965	0.911	1.022	0.228	0.236
Ruminococcaceae spp.	Adjusted for age	0.890	0.835	0.948	0.000	0.002
Ruminococcaceae spp.	Adjusted for age. sex. BMI. smoking. alcohol	0.898	0.841	0.958	0.001	0.010
Subdoligranulum spp.	Adjusted for age	0.900	0.849	0.956	0.001	0.002
Subdoligranulum spp.	Adjusted for age. sex. BMI. smoking. alcohol	0.919	0.864	0.979	0.008	0.021
Butyricicoccus spp.	Adjusted for age	0.941	0.886	0.998	0.044	0.046
Butyricicoccus spp.	Adjusted for age. sex. BMI. smoking. alcohol	0.936	0.881	0.995	0.035	0.051
Ruminococcaceae spp..1	Adjusted for age	0.928	0.890	0.967	0.000	0.002
Ruminococcaceae spp..1	Adjusted for age. sex. BMI. smoking. alcohol	0.931	0.892	0.972	0.001	0.010
Ruminococcaceae spp..2	Adjusted for age	0.877	0.812	0.948	0.001	0.003
Ruminococcaceae spp..2	Adjusted for age. sex. BMI. smoking. alcohol	0.902	0.832	0.978	0.012	0.023
[Eubacterium] coprostanoligenes group spp.	Adjusted for age	0.868	0.796	0.945	0.001	0.004
[Eubacterium] coprostanoligenes group spp.	Adjusted for age. sex. BMI. smoking. alcohol	0.885	0.809	0.965	0.006	0.018
Ruminococcus bicirculans	Adjusted for age	0.915	0.872	0.960	0.000	0.002
Ruminococcus bicirculans	Adjusted for age. sex. BMI. smoking. alcohol	0.925	0.881	0.972	0.002	0.011
[Eubacterium] coprostanoligenes group spp..1	Adjusted for age	0.944	0.898	0.991	0.020	0.028
[Eubacterium] coprostanoligenes group spp..1	Adjusted for age. sex. BMI. smoking. alcohol	0.931	0.884	0.979	0.005	0.016
Family XIII AD3011 group spp.	Adjusted for age	0.889	0.800	0.989	0.030	0.034
Family XIII AD3011 group spp.	Adjusted for age. sex. BMI. smoking. alcohol	0.921	0.825	1.027	0.139	0.149
Family XIII UCG-001 spp.	Adjusted for age	0.915	0.845	0.990	0.028	0.032
Family XIII UCG-001 spp.	Adjusted for age. sex. BMI. smoking. alcohol	0.897	0.826	0.973	0.009	0.023
Terrisporobacter mayombei	Adjusted for age	0.926	0.865	0.990	0.026	0.032
Terrisporobacter mayombei	Adjusted for age. sex. BMI. smoking. alcohol	0.934	0.870	1.000	0.054	0.070
Sutterella massiliensis/stercoricanis/wadsworthensis	Adjusted for age	1.060	1.005	1.117	0.032	0.036
Sutterella massiliensis/stercoricanis/wadsworthensis	Adjusted for age. sex. BMI. smoking. alcohol	1.049	0.993	1.107	0.086	0.098
Haemophilus spp.	Adjusted for age	1.073	1.008	1.141	0.025	0.031
Haemophilus spp.	Adjusted for age. sex. BMI. smoking. alcohol	1.060	0.994	1.130	0.072	0.084
Alistipes shahii	Adjusted for age	0.922	0.860	0.989	0.023	0.030
Alistipes shahii	Adjusted for age. sex. BMI. smoking. alcohol	0.945	0.879	1.017	0.132	0.143

Alistipes obesi	Adjusted for age	0.865	0.803	0.931	0.000	0.001
Alistipes obesi	Adjusted for age. sex. BMI. smoking. alcohol	0.905	0.837	0.977	0.011	0.023
Bacteroides caccae	Adjusted for age	0.930	0.880	0.984	0.011	0.017
Bacteroides caccae	Adjusted for age. sex. BMI. smoking. alcohol	0.947	0.895	1.003	0.063	0.079
Akkermansia muciniphila	Adjusted for age	0.938	0.894	0.983	0.009	0.015
Akkermansia muciniphila	Adjusted for age. sex. BMI. smoking. alcohol	0.961	0.915	1.009	0.113	0.125
Bifidobacterium spp.	Adjusted for age	1.066	1.017	1.118	0.008	0.014
Bifidobacterium spp.	Adjusted for age. sex. BMI. smoking. alcohol	1.062	1.012	1.116	0.015	0.027
Eggerthellaceae spp.	Adjusted for age	0.917	0.862	0.975	0.006	0.011
Eggerthellaceae spp.	Adjusted for age. sex. BMI. smoking. alcohol	0.927	0.869	0.988	0.020	0.033
Enterorhabdus spp.	Adjusted for age	0.941	0.888	0.996	0.036	0.039
Enterorhabdus spp.	Adjusted for age. sex. BMI. smoking. alcohol	0.941	0.887	0.999	0.048	0.064
Streptococcus spp.	Adjusted for age	1.083	1.014	1.157	0.018	0.026
Streptococcus spp.	Adjusted for age. sex. BMI. smoking. alcohol	1.063	0.994	1.137	0.073	0.084
Streptococcus spp..1	Adjusted for age	1.174	1.091	1.263	0.000	0.000
Streptococcus spp..1	Adjusted for age. sex. BMI. smoking. alcohol	1.133	1.051	1.221	0.001	0.010
Catenibacterium mitsuokai	Adjusted for age	1.046	1.005	1.089	0.026	0.032
Catenibacterium mitsuokai	Adjusted for age. sex. BMI. smoking. alcohol	1.016	0.974	1.059	0.469	0.469
Erysipelotrichaceae UCG-003 bacterium	Adjusted for age	0.917	0.866	0.970	0.002	0.006
Erysipelotrichaceae UCG-003 bacterium	Adjusted for age. sex. BMI. smoking. alcohol	0.950	0.895	1.008	0.089	0.100
Clostridia spp.	Adjusted for age	0.900	0.849	0.953	0.000	0.002
Clostridia spp.	Adjusted for age. sex. BMI. smoking. alcohol	0.921	0.867	0.976	0.006	0.017
Clostridia spp..1	Adjusted for age	0.901	0.840	0.964	0.003	0.007
Clostridia spp..1	Adjusted for age. sex. BMI. smoking. alcohol	0.912	0.848	0.977	0.010	0.023
Clostridia spp..2	Adjusted for age	0.934	0.879	0.991	0.026	0.031
Clostridia spp..2	Adjusted for age. sex. BMI. smoking. alcohol	0.956	0.898	1.017	0.159	0.166
Clostridium sensu stricto 1 spp.	Adjusted for age	0.917	0.853	0.985	0.018	0.026
Clostridium sensu stricto 1 spp.	Adjusted for age. sex. BMI. smoking. alcohol	0.913	0.847	0.981	0.014	0.027
Clostridium sensu stricto 1 spp..1	Adjusted for age	0.908	0.858	0.960	0.001	0.003
Clostridium sensu stricto 1 spp..1	Adjusted for age. sex. BMI. smoking. alcohol	0.912	0.860	0.965	0.002	0.010
Clostridium sensu stricto 1 celatum/disporicum/saudiense	Adjusted for age	0.937	0.882	0.994	0.031	0.035
Clostridium sensu stricto 1 celatum/disporicum/saudiense	Adjusted for age. sex. BMI. smoking. alcohol	0.927	0.872	0.985	0.015	0.027
Christensenellaceae R-7 group spp.	Adjusted for age	0.864	0.788	0.946	0.002	0.005
Christensenellaceae R-7 group spp.	Adjusted for age. sex. BMI. smoking. alcohol	0.879	0.799	0.964	0.007	0.019
Christensenellaceae R-7 group spp..1	Adjusted for age	0.912	0.851	0.978	0.010	0.016
Christensenellaceae R-7 group spp..1	Adjusted for age. sex. BMI. smoking. alcohol	0.918	0.854	0.987	0.020	0.033
Christensenellaceae R-7 group spp..2	Adjusted for age	0.909	0.855	0.967	0.002	0.006

Christensenellaceae R-7 group spp..2	Adjusted for age. sex. BMI. smoking. alcohol	0.913	0.857	0.973	0.005	0.016
Christensenellaceae R-7 group spp..3	Adjusted for age	0.893	0.820	0.971	0.009	0.016
Christensenellaceae R-7 group spp..3	Adjusted for age. sex. BMI. smoking. alcohol	0.891	0.815	0.971	0.009	0.023
Christensenellaceae R-7 group spp..4	Adjusted for age	0.882	0.841	0.925	0.000	0.000
Christensenellaceae R-7 group spp..4	Adjusted for age. sex. BMI. smoking. alcohol	0.897	0.853	0.943	0.000	0.001
Clostridia spp..3	Adjusted for age	0.878	0.808	0.951	0.002	0.005
Clostridia spp..3	Adjusted for age. sex. BMI. smoking. alcohol	0.880	0.809	0.956	0.003	0.012
Clostridia UCG-014 spp.	Adjusted for age	0.869	0.810	0.931	0.000	0.001
Clostridia UCG-014 spp.	Adjusted for age. sex. BMI. smoking. alcohol	0.885	0.823	0.950	0.001	0.010
Monoglobus spp.	Adjusted for age	0.885	0.821	0.955	0.002	0.004
Monoglobus spp.	Adjusted for age. sex. BMI. smoking. alcohol	0.910	0.842	0.984	0.017	0.029
[Eubacterium] hallii group spp.	Adjusted for age	0.899	0.824	0.982	0.017	0.025
[Eubacterium] hallii group spp.	Adjusted for age. sex. BMI. smoking. alcohol	0.913	0.834	1.002	0.051	0.068
[Eubacterium] hallii group spp..1	Adjusted for age	0.927	0.860	0.999	0.045	0.046
[Eubacterium] hallii group spp..1	Adjusted for age. sex. BMI. smoking. alcohol	0.926	0.858	1.001	0.052	0.068
Lachnospiraceae spp..6	Adjusted for age	0.821	0.757	0.890	0.000	0.000
Lachnospiraceae spp..6	Adjusted for age. sex. BMI. smoking. alcohol	0.843	0.775	0.916	0.000	0.002
[Ruminococcus] gnavus group spp.	Adjusted for age	1.069	1.007	1.134	0.027	0.032
[Ruminococcus] gnavus group spp.	Adjusted for age. sex. BMI. smoking. alcohol	1.072	1.006	1.140	0.029	0.043
Marvinbryantia spp..1	Adjusted for age	0.852	0.781	0.928	0.000	0.002
Marvinbryantia spp..1	Adjusted for age. sex. BMI. smoking. alcohol	0.863	0.788	0.944	0.001	0.010
Marvinbryantia spp..2	Adjusted for age	0.887	0.800	0.983	0.022	0.029
Marvinbryantia spp..2	Adjusted for age. sex. BMI. smoking. alcohol	0.896	0.806	0.998	0.044	0.062
Blautia stercoris	Adjusted for age	1.061	1.000	1.124	0.048	0.048
Blautia stercoris	Adjusted for age. sex. BMI. smoking. alcohol	1.049	0.987	1.114	0.117	0.128
Blautia obeum	Adjusted for age	0.903	0.831	0.982	0.017	0.025
Blautia obeum	Adjusted for age. sex. BMI. smoking. alcohol	0.922	0.845	1.008	0.071	0.084
Blautia spp.	Adjusted for age	0.899	0.829	0.975	0.010	0.016
Blautia spp.	Adjusted for age. sex. BMI. smoking. alcohol	0.894	0.823	0.973	0.009	0.023
Blautia spp..1	Adjusted for age	0.841	0.765	0.924	0.000	0.002
Blautia spp..1	Adjusted for age. sex. BMI. smoking. alcohol	0.859	0.778	0.947	0.002	0.012
Lachnospiraceae spp..7	Adjusted for age	0.915	0.865	0.968	0.002	0.005
Lachnospiraceae spp..7	Adjusted for age. sex. BMI. smoking. alcohol	0.935	0.881	0.991	0.024	0.037
Lachnospiraceae spp..8	Adjusted for age	0.867	0.784	0.959	0.005	0.011
Lachnospiraceae spp..8	Adjusted for age. sex. BMI. smoking. alcohol	0.872	0.786	0.969	0.010	0.023
Lachnospiraceae spp..9	Adjusted for age	0.929	0.867	0.993	0.033	0.036
Lachnospiraceae spp..9	Adjusted for age. sex. BMI. smoking. alcohol	0.934	0.870	1.001	0.056	0.071

Roseburia hominis	Adjusted for age	0.900	0.835	0.970	0.006	0.012
Roseburia hominis	Adjusted for age. sex. BMI. smoking. alcohol	0.914	0.846	0.987	0.021	0.034
Lachnospiraceae spp..10	Adjusted for age	0.897	0.838	0.958	0.001	0.004
Lachnospiraceae spp..10	Adjusted for age. sex. BMI. smoking. alcohol	0.902	0.842	0.965	0.003	0.012

Table shows coefficients resulting from logistic regression models for incident hypertension. including the odds ratios (OR) and 95%-confidence intervals (CI). P-values were adjusted using the false discovery ratio. All amplicon sequence variants (ASVs) that were significant after adjustment are listed. A numeric suffix was appended to features with identical taxonomic classifications to ensure unique names.

Supplementary Table 9: Subgroup with serum metabolomics data

	**Overall**	**Dutch**	**South-Asian Surinamese**	**p**
**n**	105	51	54	
**Age (years)**	52.0 (10.5)	51.5 (11.2)	52.6 (9.8)	0.573
**Women**	63 (60.0)	30 (58.8)	33 (61.1)	0.968
**BMI (kg/m2)**	25.6 (3.7)	25.7 (3.9)	25.5 (3.6)	0.791
**Smoking**				<0.001
Yes	17 (16.2)	7 (13.7)	10 (18.5)	
Never	54 (51.4)	18 (35.3)	36 (66.7)	
Former smoking	34 (32.4)	26 (51.0)	8 (14.8)	
**Diabetes**	5 (4.8)	2 (3.9)	3 (5.6)	1.000
**Systolic BP (mmHg)**	128.3 (19.9)	127.0 (20.5)	129.7 (19.3)	0.488
**Diastolic BP (mmHg)**	78.9 (10.8)	77.7 (10.5)	80.0 (11.0)	0.278
**Hypertension**	36 (34.3)	17 (33.3)	19 (35.2)	1.000
**Dyslipidemia**	29 (27.6)	8 (15.7)	21 (38.9)	0.015
**Total cholesterol (mmol/L)**	5.2 (0.9)	5.3 (0.9)	5.2 (0.8)	0.309
**LDL (mmol/L)**	3.3 (0.8)	3.3 (0.8)	3.3 (0.8)	0.955
**Triglycerides (mmol/L)**	0.9 [0.6, 1.2]	0.8 [0.6, 1.2]	0.9 [0.7, 1.3]	0.129
**HbA1c (mmol/mol)**	38.9 (6.0)	36.5 (3.9)	41.3 (6.8)	<0.001

Data is presented as mean±SD, median [interquartile range] or n (%). BMI = body mass index, BP = blood pressure, LDL = low-density lipoprotein.

Supplementary Table 10: STROBE checklist

	Item No	Recommendation	Page number
**Title and abstract**	1	(*a*) Indicate the study's design with a commonly used term in the title or the abstract	2
		(*b*) Provide in the abstract an informative and balanced summary of what was done and what was found	2
**Introduction**			
Background/rationale	2	Explain the scientific background and rationale for the investigation being reported	4
Objectives	3	State specific objectives, including any prespecified hypotheses	4
**Methods**			
Study design	4	Present key elements of study design early in the paper	5
Setting	5	Describe the setting, locations, and relevant dates, including periods of recruitment, exposure, follow-up, and data collection	5,7
Participants	6	(*a*) Give the eligibility criteria, and the sources and methods of selection of participants. Describe methods of follow-up	5,6
		(*b*) For matched studies, give matching criteria and number of exposed and unexposed	8
Variables	7	Clearly define all outcomes, exposures, predictors, potential confounders, and effect modifiers. Give diagnostic criteria, if applicable	6,7
Data sources/ measurement	8*	For each variable of interest, give sources of data and details of methods of assessment (measurement). Describe comparability of assessment methods if there is more than one group	5,6,7
Bias	9	Describe any efforts to address potential sources of bias	9
Study size	10	Explain how the study size was arrived at	5
Quantitative variables	11	Explain how quantitative variables were handled in the analyses. If applicable, describe which groupings were chosen and why	8,9
Statistical methods	12	(*a*) Describe all statistical methods, including those used to control for confounding	8,9
		(*b*) Describe any methods used to examine subgroups and interactions	9
		(*c*) Explain how missing data were addressed	9
		(*d*) If applicable, explain how loss to follow-up was addressed	9
		(*e*) Describe any sensitivity analyses	9
**Results**			
Participants	13*	(a) Report numbers of individuals at each stage of study—eg numbers potentially eligible, examined for eligibility, confirmed eligible, included in the study, completing follow-up, and analysed	5-9
		(b) Give reasons for non-participation at each stage	5-9
		(c) Consider use of a flow diagram	
Descriptive data	14*	(a) Give characteristics of study participants (eg demographic, clinical, social) and information on exposures and potential confounders	27
		(b) Indicate number of participants with missing data for each variable of interest	9
		(c) Summarise follow-up time (eg, average and total amount)	27
Outcome data	15*	Report numbers of outcome events or summary measures over time	11, 27
Main results	16	(*a*) Give unadjusted estimates and, if applicable, confounder-adjusted estimates and their precision (eg, 95% confidence interval). Make clear which confounders were adjusted for and why they were included	11-13
		(*b*) Report category boundaries when continuous variables were categorized	NA

		(*c*) If relevant, consider translating estimates of relative risk into absolute risk for a meaningful time period	NA
Other analyses	17	Report other analyses done—eg analyses of subgroups and interactions, and sensitivity analyses	12-14
**Discussion**			
Key results	18	Summarise key results with reference to study objectives	16
Limitations	19	Discuss limitations of the study, taking into account sources of potential bias or imprecision. Discuss both direction and magnitude of any potential bias	19-20
Interpretation	20	Give a cautious overall interpretation of results considering objectives, limitations, multiplicity of analyses, results from similar studies, and other relevant evidence	20
Generalisability	21	Discuss the generalisability (external validity) of the study results	19-20
**Other information**			
Funding	22	Give the source of funding and the role of the funders for the present study and, if applicable, for the original study on which the present article is based	22

*Give information separately for exposed and unexposed groups.

Supplementary Table 11: Major adverse cardiovascular event definition

Major adverse cardiovascular event definition

Fatal endpoints

**Included mortality causes**	**ICD-10**
Hypertensive disease	I10-16
Ischemic heart disease	I20-25
Arrhythmias, heart failure	I46-52
Cerebrovascular disease	I60-69
Atherosclerosis/AAA	I70-73
Sudden death and death within 24h of symptom onset	R96.0-96.1

**Excluded mortality causes**	**ICD-10**
Myocarditis, unspecified	I51.4
Subarachnoid hemorrhage	I60
Subdural hemorrhage	I62
Cerebral aneurysm	I67.1
Cerebral arteritis	I68.2
Moyamoya	I67.5

Non-fatal endpoints

**Included hospitalizations**	**ICD-10**
Non-fatal myocardial infarction	I21-I23
Non-fatal stroke	I60-I69

**Excluded hospitalizations**	**ICD-10**
Subarachnoid hemorrhage	I60
Subdural hemorrhage	I62
Cerebral aneurysm	I67.1
Cerebral arteritis	I68.2
Moyamoya	I67.5

Additionally, we included coronary revascularizations that were not labeled with the ICD-codes above.

Additionally included for MACE-plus

**Included hospitalizations**	**ICD-10**
Angina pectoris	I20

Supplementary Figure 1: Rarefaction curve

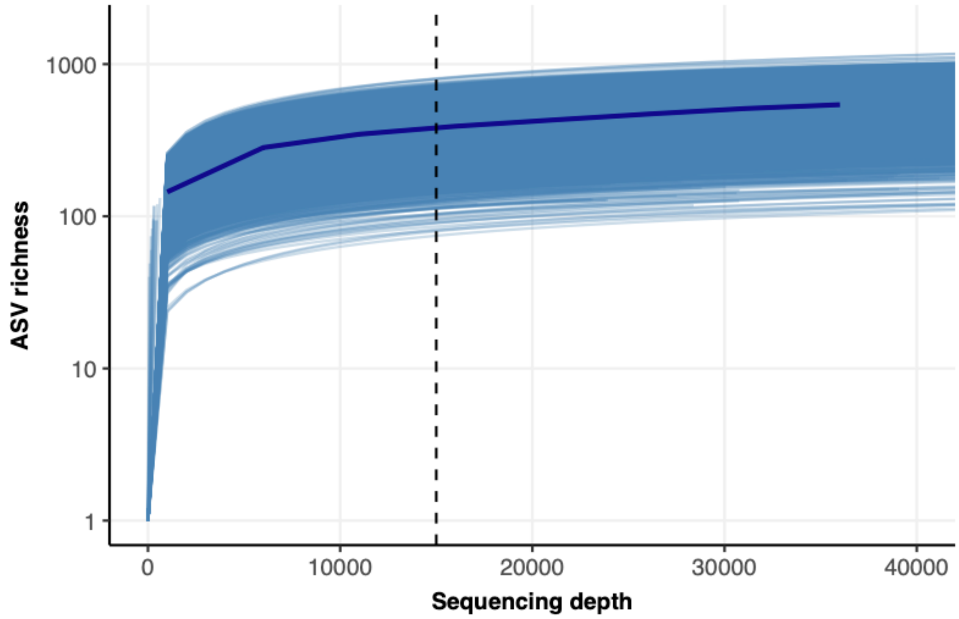

Rarefaction curve of the microbiome data. The rarefaction threshold of 15.000 counts was chosen since this threshold was well above the point of plateauing of the rarefaction curve while not excluding too many samples that fell under this threshold (14 samples).

Supplementary Figure 2: Cox regression models for MACE stratified for ethnicity

A

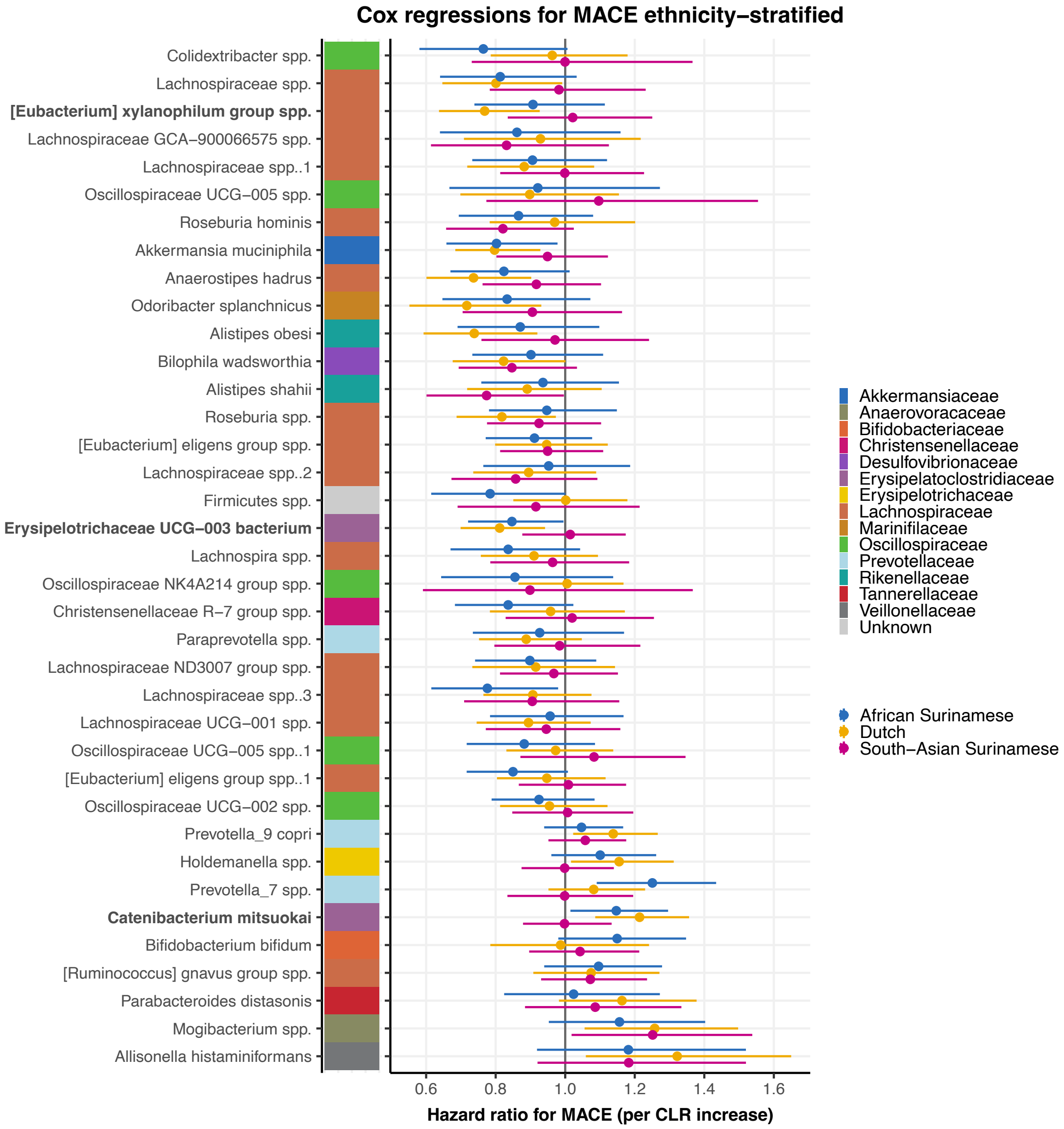

B

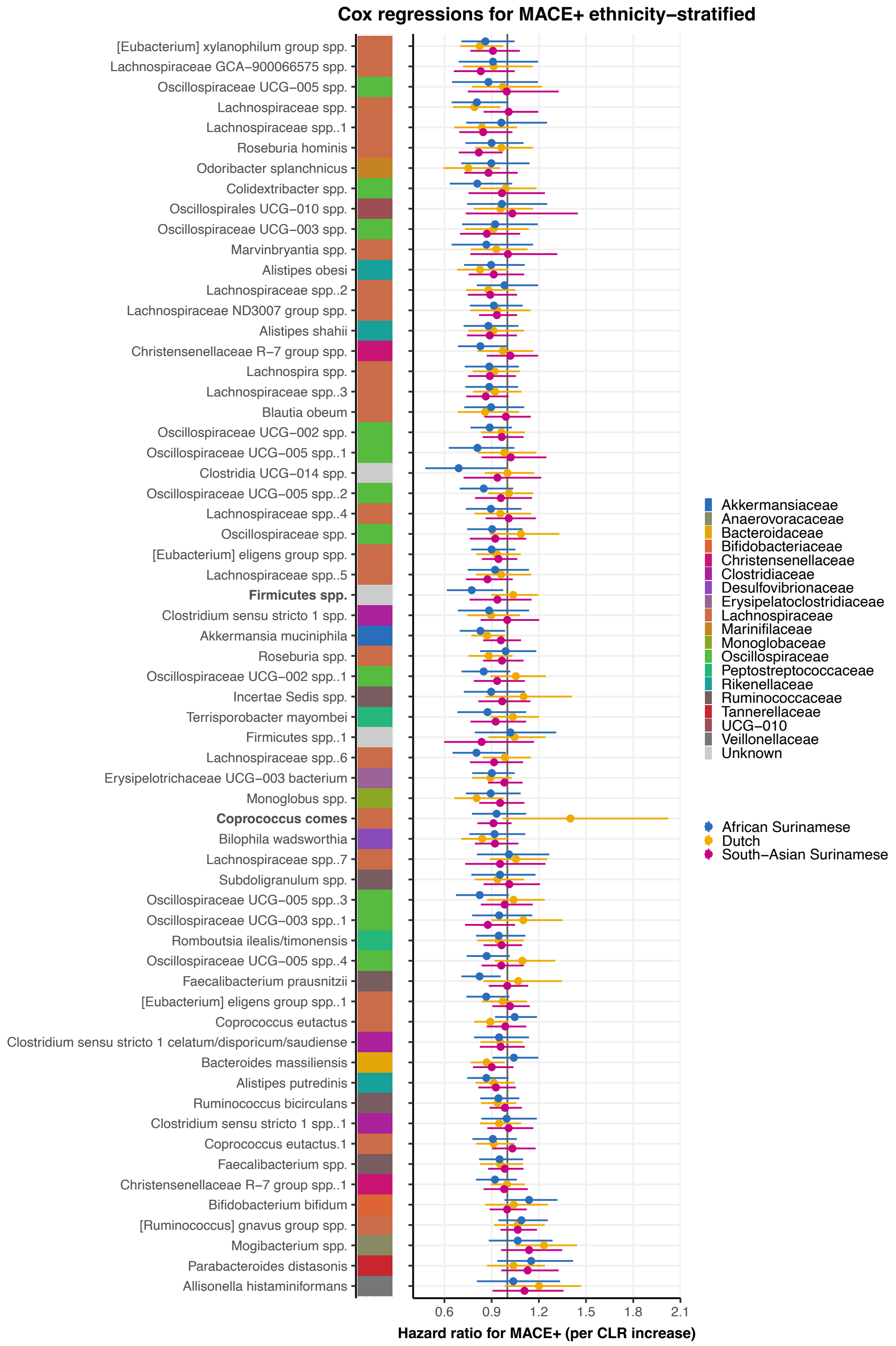

Cox regression models between baseline gut microbes and MACE. stratified for ethnicity. *Regression models per outcome: MACE (A) and MACE+. additionally including hospitalizations for angina pectoris (B). Forest plots with estimates (hazard ratios) and 95% confidence intervals per centered log-ratio (CLR) increase in the amplicon sequence variant (ASV). The colored bar next to the taxonomic name of the ASV indicates the phylogenetic family. The ASVs with significant interactions with ethnicity are shown in bold font. A numeric suffix was appended to features with identical taxonomic classifications to ensure unique names.*

Supplementary Figure 3: Cox regression models for MACE stratified for sex

A

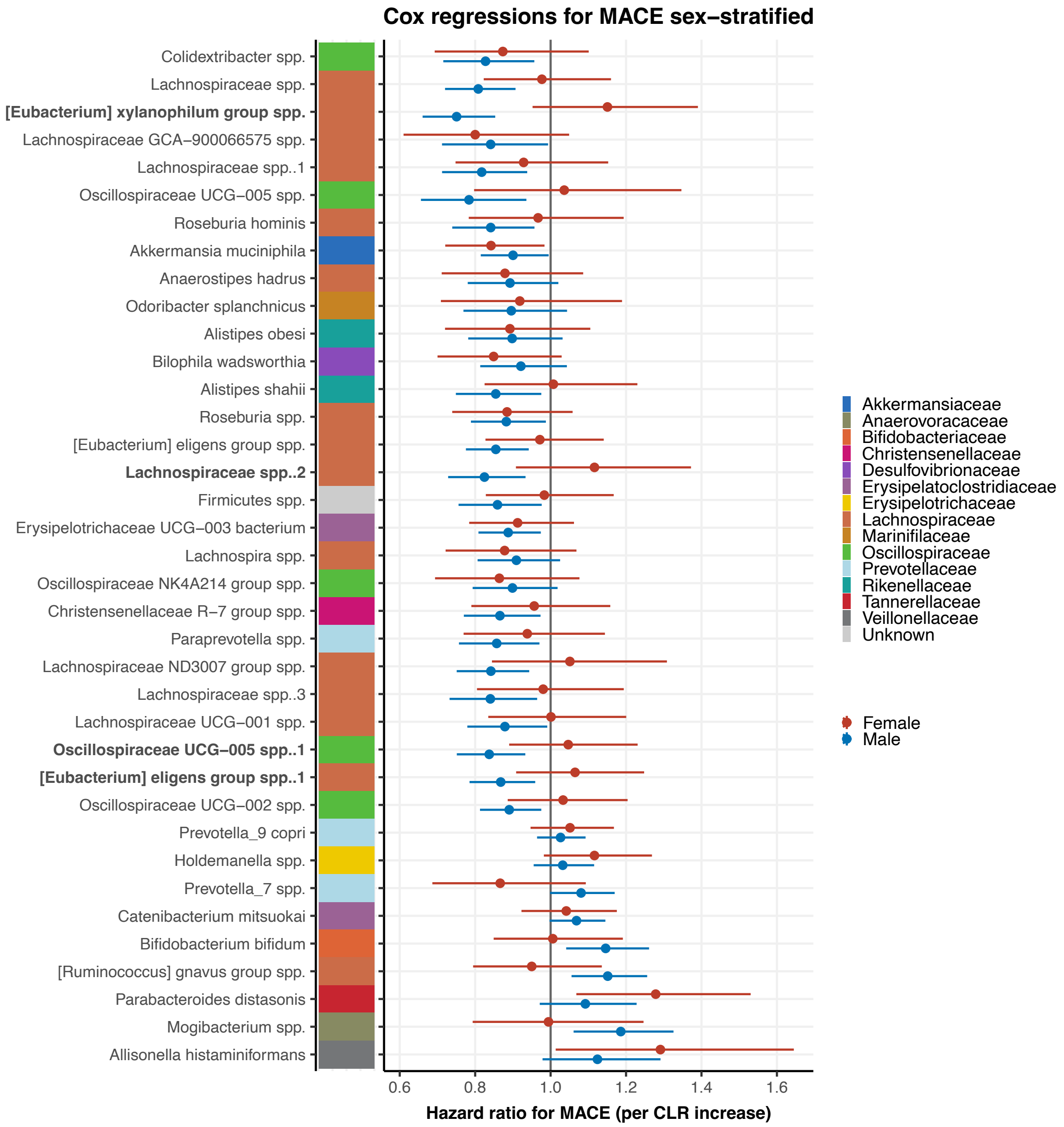

B

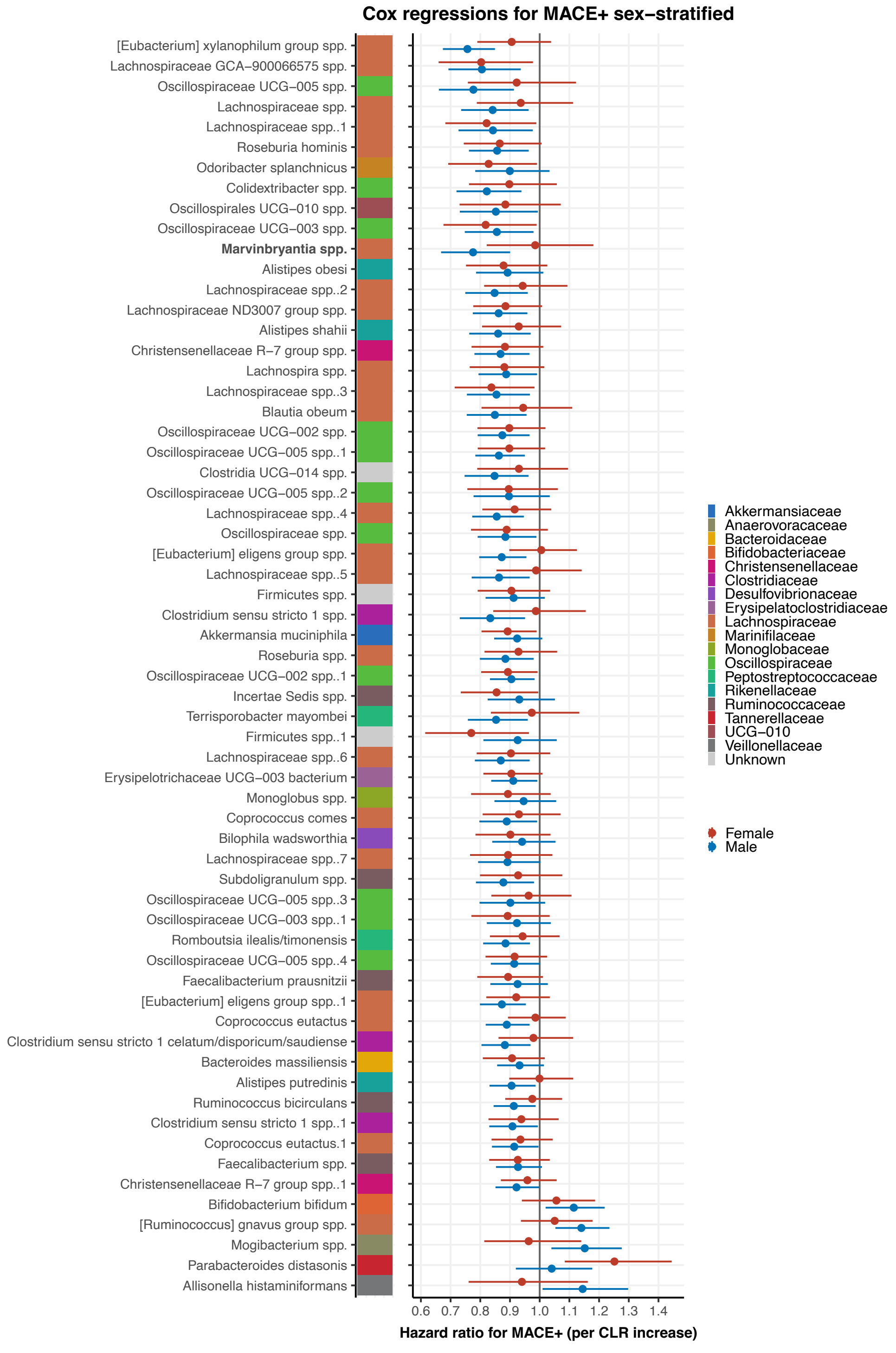

Cox regression models between baseline gut microbes and MACE. stratified for sex. *Regression models per outcome: MACE (A) and MACE+. additionally including hospitalizations for angina pectoris (B). Forest plots with estimates (hazard ratios) and 95% confidence intervals per centered log-ratio increase in the amplicon sequence variant (ASV). The colored bar next to the taxonomic name of the ASV indicates the phylogenetic family. The ASVs with significant interactions with sex are shown in bold font. A numeric suffix was appended to features with identical taxonomic classifications to ensure unique names.*

Supplementary Figure 4: Competing risk analyses

A

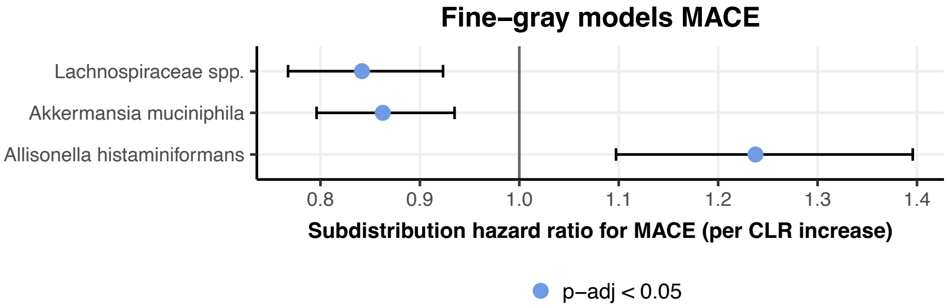

B

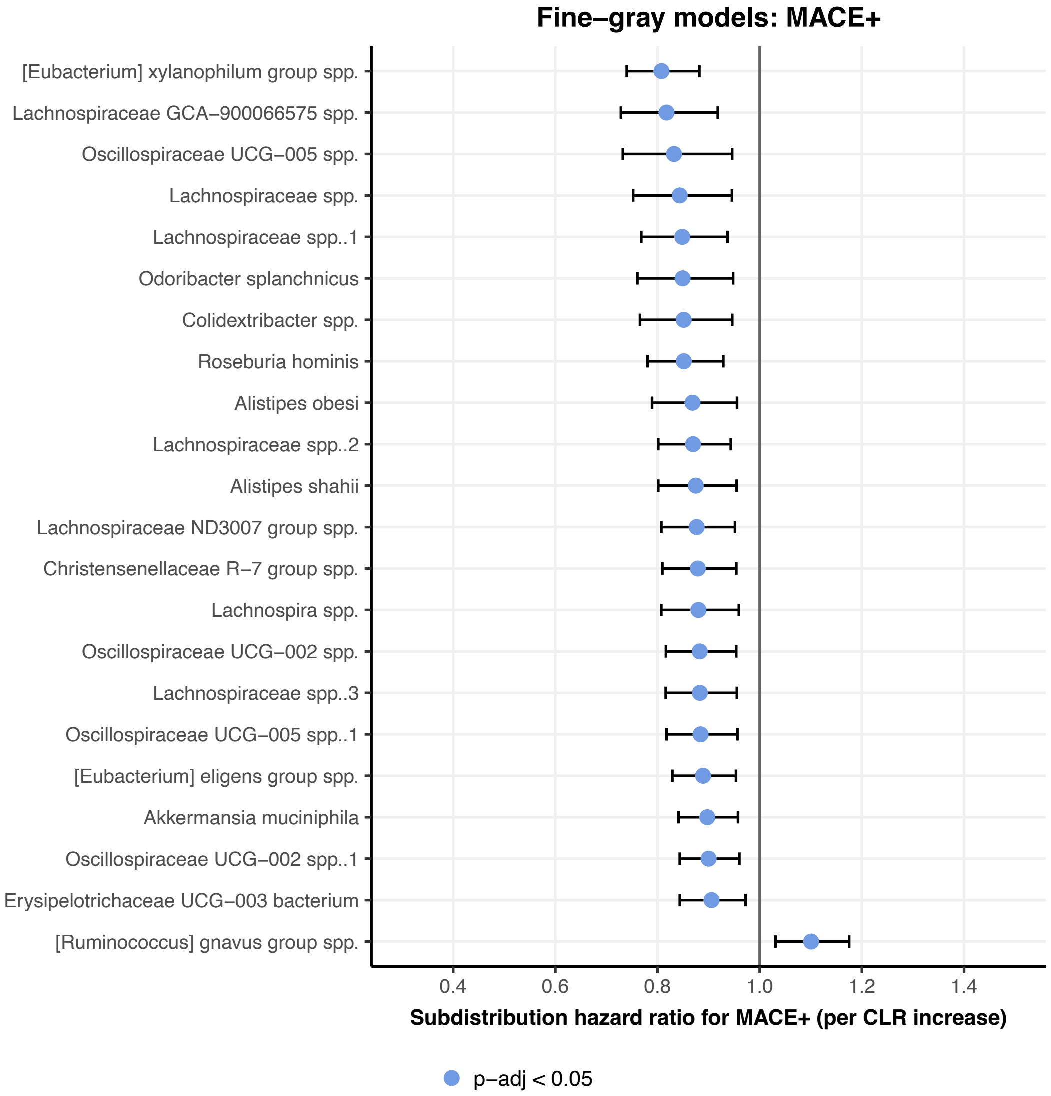

Fine-gray models between baseline gut microbes and MACE with non-cardiovascular death as competing risk. *Regression models per outcome: MACE (A) and MACE+. additionally including hospitalizations for angina pectoris (B); Forest plots with estimates (subdistribution hazard ratios) and 95% confidence intervals per centered log-ratio increase in the amplicon sequence variant (ASV). A numeric suffix was appended to features with identical taxonomic classifications to ensure unique names.*

Supplementary Figure 5: Logistic regressions incident cardiometabolic diagnoses stratified for ethnicity

A

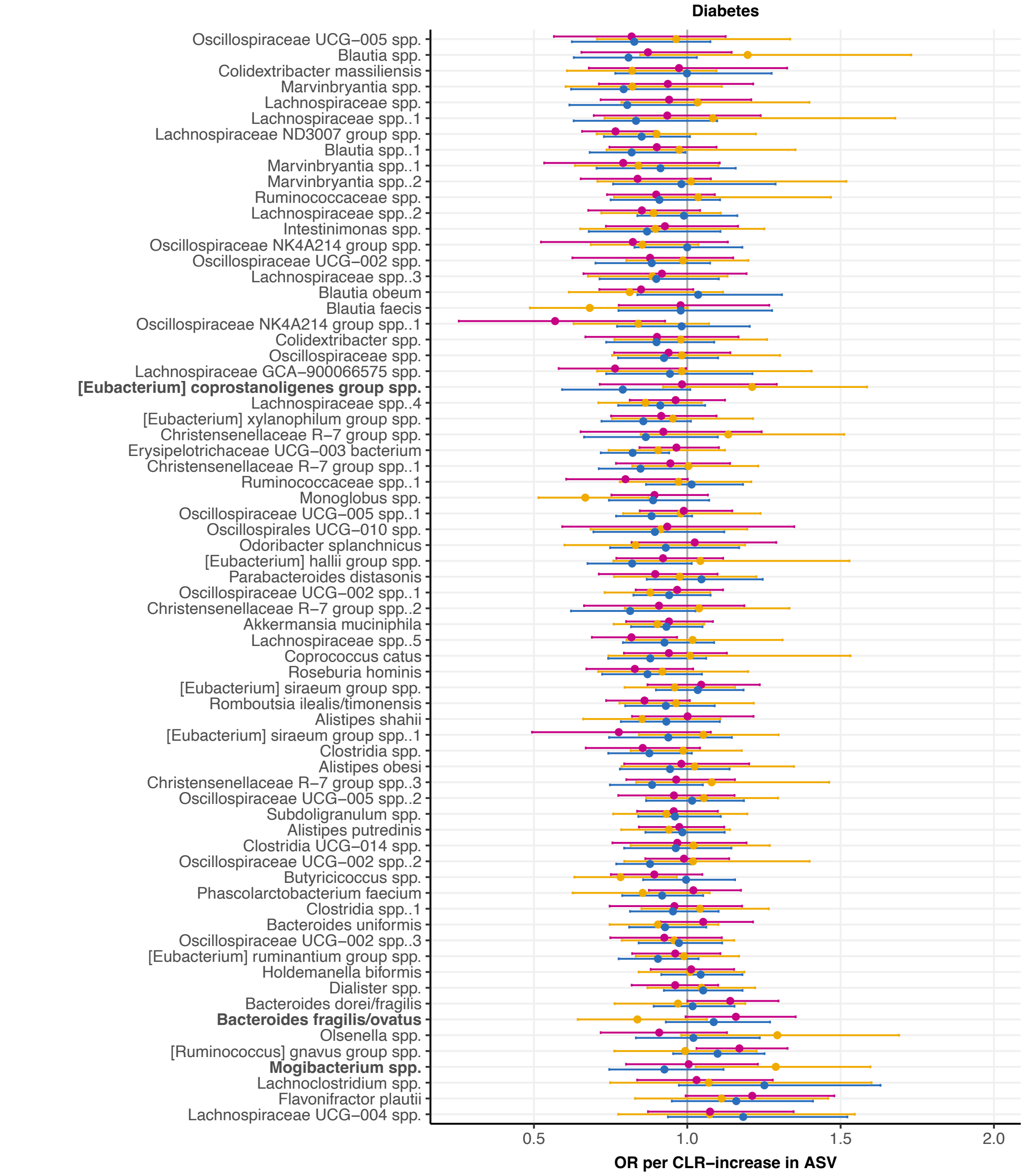

B

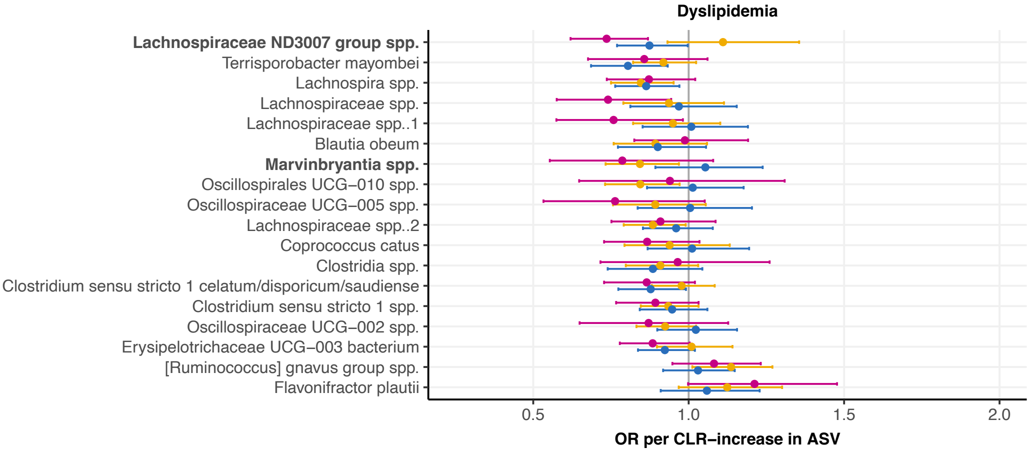

C

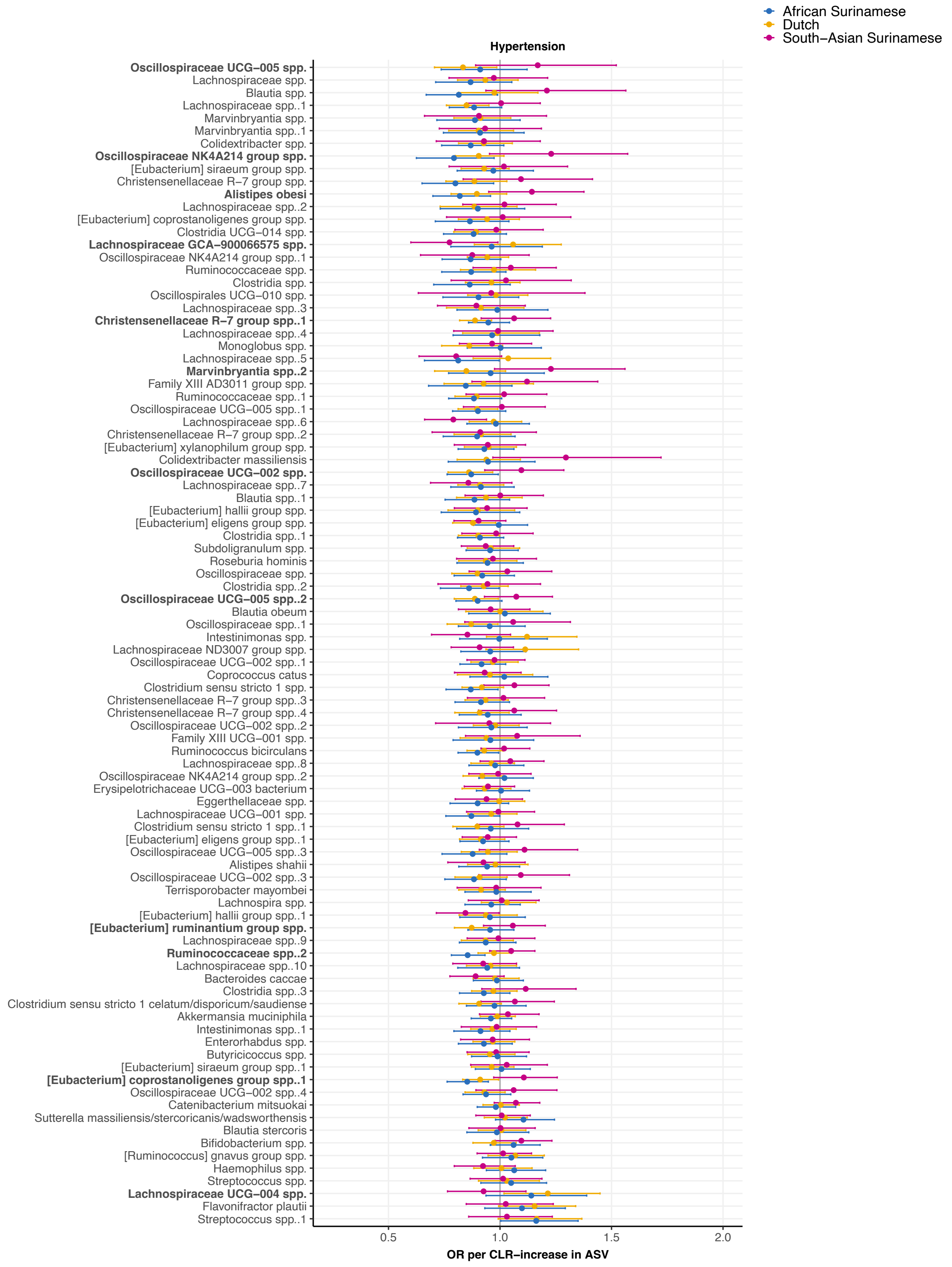

Logistic regression models between baseline gut microbes and incident cardiometabolic diagnoses. stratified for ethnicity. *Regression models per outcome: Diabetes (A) and dyslipidemia (B) and hypertension (C); Forest plots with estimates (odds ratios) and 95% confidence intervals per centered log-ratio increase in the amplicon sequence variant (ASV). The ASVs with significant interactions with ethnicity are shown in bold font. A numeric suffix was appended to features with identical taxonomic classifications to ensure unique names.*

Supplementary Figure 6: Logistic regressions incident cardiometabolic diagnoses stratified for sex

A

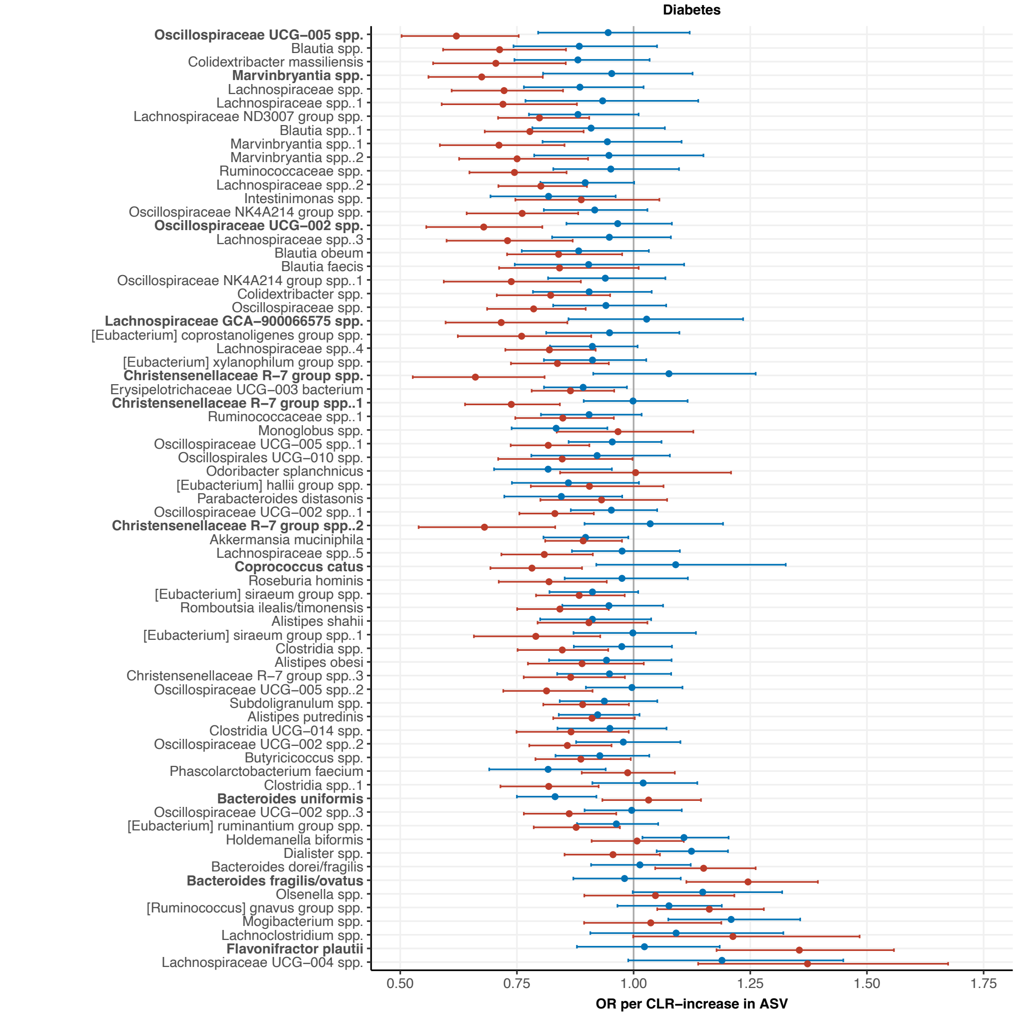

B

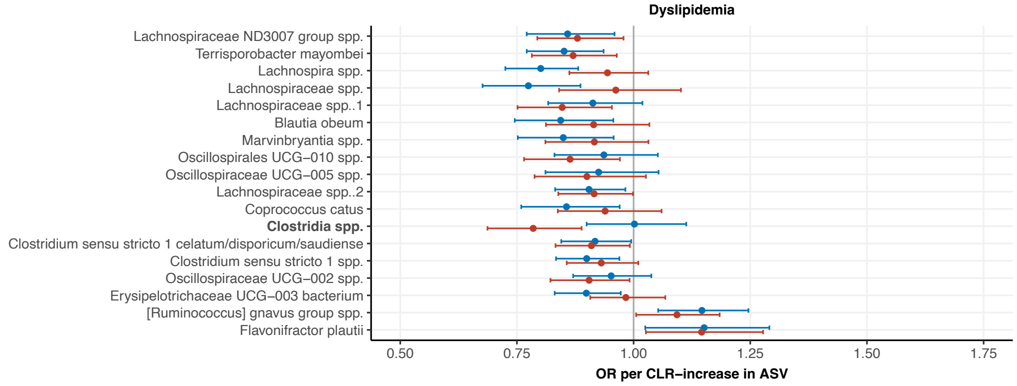

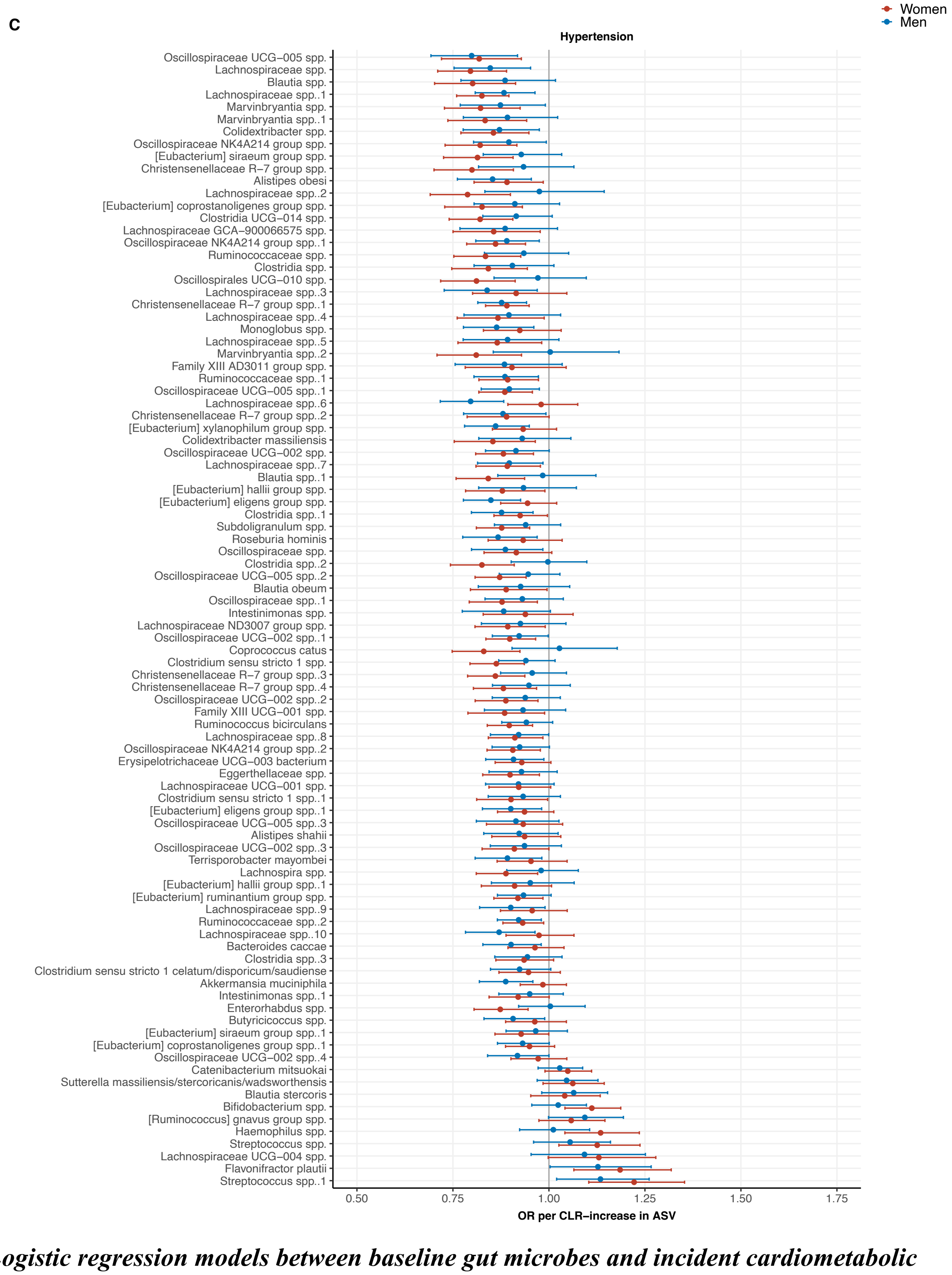

Logistic regression models between baseline gut microbes and incident cardiometabolic diagnoses. stratified for sex. *Regression models per outcome: Diabetes (A) and dyslipidemia (B) and hypertension (C); Forest plots with estimates (odds ratios) and 95% confidence intervals per centered log-ratio increase in the amplicon sequence variant (ASV). The ASVs with significant interactions with sex are shown in bold font. A numeric suffix was appended to features with identical taxonomic classifications to ensure unique names.*

Supplementary Figure 7: Correlation plots microbes and metabolites

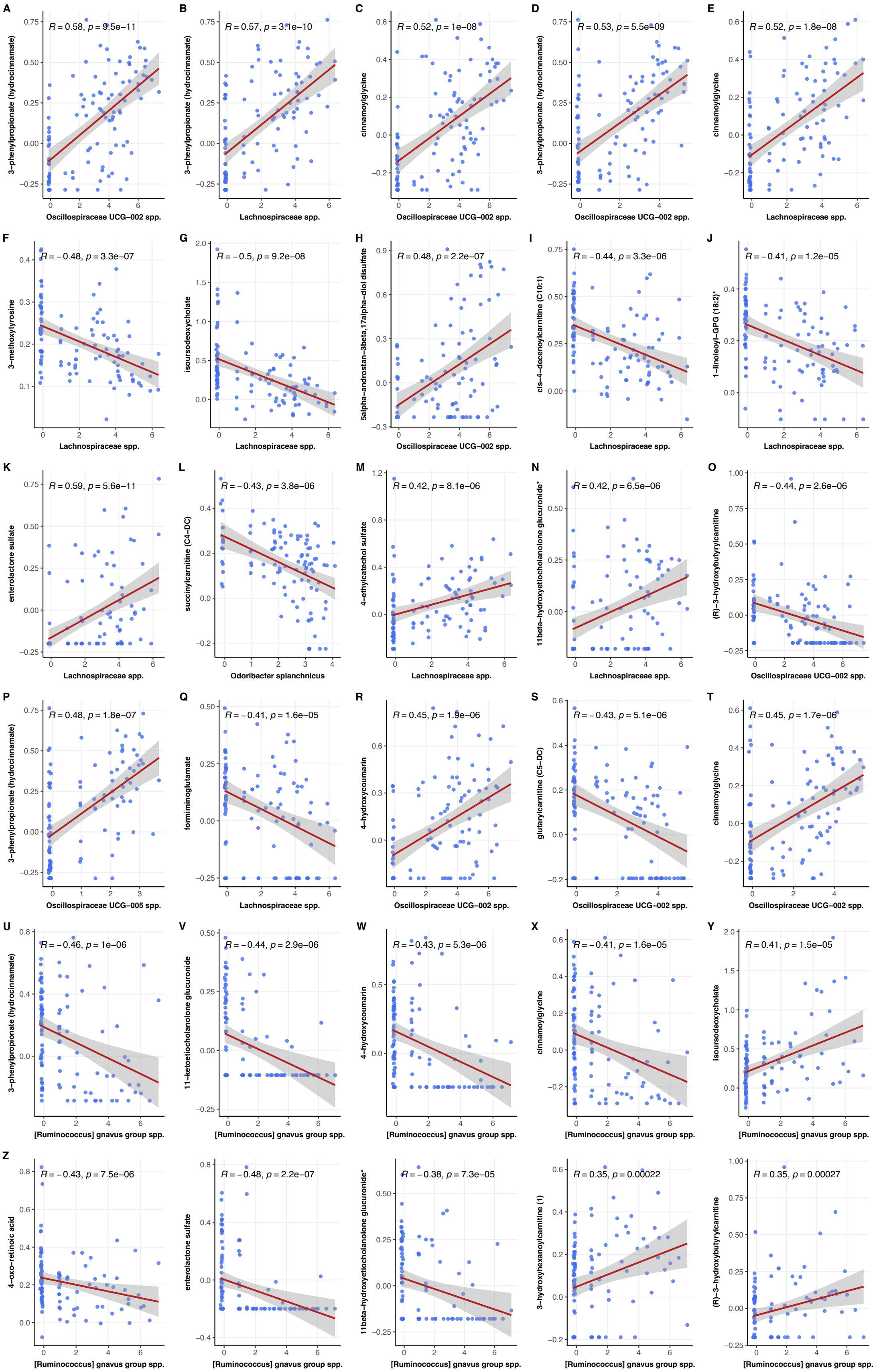

Correlation plots of microbe abundances and metabolites in 105 participants. *Correlation plots (Spearman's coefficients) showing the 30 strongest correlations between MACE-predicting amplicon sequence variants (ASVs) and serum metabolites.*
